# Modified Kumaraswamy seasonal autoregressive moving average models with exogenous regressors for double-bounded hydro-environmental data

**DOI:** 10.1371/journal.pone.0324721

**Published:** 2025-05-20

**Authors:** Aline Armanini Stefanan, Murilo Sagrillo, Bruna G. Palm, Fábio M. Bayer

**Affiliations:** 1 Postgraduate Program in Industrial Engineering, Universidade Federal de Santa Maria, Santa Maria, Rio Grande do Sul, Brazil; 2 Department of Mathematics and Natural Sciences, Blekinge Institute of Technology, Karlskrona, Blekinge, Sweden; 3 Department of Statistics, Universidade Federal de Santa Maria, Santa Maria, Rio Grande do Sul, Brazil; University of Queensland - Saint Lucia Campus: The University of Queensland, AUSTRALIA

## Abstract

This paper proposes the MKSARMAX model for modeling and forecasting time series that can only take on values within a specified range, such as in the interval (0,1). The model is especially good for modeling double-bounded hydro-environmental time series since it accommodates bounded support and asymmetric distribution, making it advantageous compared to the traditional Gaussian-based time series model. The MKSARMAX models the conditional median of a modified Kumaraswamy distributed variable observed over time, by a dynamic structure considering stochastic seasonality and including autoregressive and moving average terms, exogenous regressors, and a link function. The conditional maximum likelihood method is employed to estimate the model parameters. Hypothesis tests and confidence intervals for the parameters of the proposed model are derived using the asymptotic theory of the conditional maximum likelihood estimators. Quantile residuals are defined for diagnostic analysis, and goodness-of-fit tests are subsequently implemented. Synthetic hydro-environmental time series are generated in a Monte Carlo simulation study to assess the finite sample performance of the inferences. Moreover, MKSARMAX outperforms *β*SARMA, SARMAX, Holt-Winters, and KARMA models in most accuracy measures analyzed when applied to useful water volume datasets, presenting for the first-step forecast at least 98% lower MAE, RMSE, and MAPE values than competitors in the Caconde UV dataset, and 54% lower MAE, RMSE, and MAPE values than competitors in the Guarapiranga UV dataset. These findings suggest that the MKSARMAX model holds strong potential for water resource management. Its flexibility and accuracy in the early forecasting steps make it particularly valuable for predicting flood and drought periods.

## 1 Introduction

Improving modeling capability and forecast accuracy for hydro-environmental variables remains an ongoing objective in scientific research. Hydro-environmental time series, such as relative humidity, water level, rainfall depth or volume, wave height, streamflow, and groundwater level values recorded at regular periods of time, are generally modeled from a stochastic view by the traditional Gaussian-based autoregressive moving average (ARMA) model [1], which considers the time series is real-valued and follows a mesokurtic and symmetric distribution. However, several hydro-environmental variables, such as useful water volume, are asymmetrically distributed and present bounded support [2]. In such situations, the assumption of Gaussianity can lead to inaccurate inferences, wrong predictions, and erroneous interpretations, such as predicting values out of bounds [3,4]. One approach to extending the Gaussian ARMA for non-Gaussian time series data was the generalized autoregressive moving average (GARMA) model, proposed by [5], which is an extension to the work of [6] - the autoregressive and Markov chain models for time series, and [7] - that considered the moving average component. The GARMA model has a dynamic generalized linear model (GLM) framework [8] and proposes to model data following the conditional canonical exponential family distribution, such as Poisson, binomial, and gamma distributions.

**Fig 1:**
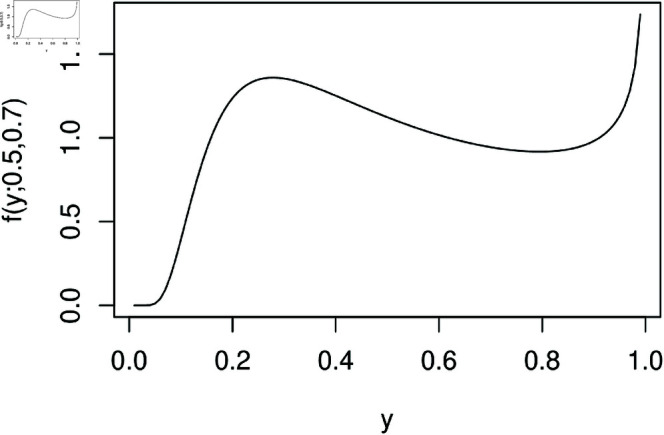
MK density for parameters α=0.5 and λ=0.7.

In a stochastic approach, some probabilistic distributions that are usually applied to hydrological processes are the Kumaraswamy[9], inflated Kumaraswamy [10], beta [11–13], inflated beta [14], beta prime [15], kappa [16], Rayleigh [17], inflated Rayleigh [18], Weibull [19], and Gumbel [20], for example. In particular, when the variable of interest exhibits double-boundedness and an asymmetric distribution, researchers have considered the beta and Kumaraswamy laws [9,13,14,21,22]. Dynamic models based on these distributions, the beta autoregressive moving average (βARMA) and the Kumaraswamy autoregressive moving average (KARMA) models, were proposed in [3] and [9], respectively. Additionally, the beta seasonal autoregressive moving average (βSARMA) model, proposed by [21], extends the class of βARMA models by incorporating seasonal dynamics. However, as discussed in [2], the beta and Kumaraswamy-based models may have limitations in their flexibility for modeling certain types of hydrological data. Thus, [2] proposed a more flexible two-parameter probability model called the modified Kumaraswamy (MK) distribution. This new probability model incorporates density shapes unsuitable for the beta and Kumaraswamy distributions, such as increasing-decreasing-increasing shapes, as can be seen in the Fig 1. Also, it can be useful to model left- or right-skewed data and heavy- or non-heavy tails.

Considering an empirical application in the percentage of useful water volume of several Brazilian water reservoirs, the authors showed that the MK distribution excels over competing models (beta and Kumaraswamy), being a suitable and prominent alternative for modeling double-bounded hydro-environmental data.

To fit the MK model, [2] assumes constant parameters and independence among the data points, which are strong assumptions in time series data. To the best of our knowledge, a dynamic model based on the MK distribution has not been addressed in the literature, and this paper aims to provide the first treatment on this topic.

### 1.1 Research motivation and objective

It is common knowledge that natural resources are finite on our planet, and since our life depends on them, it is imperative that we monitor and utilize these resources efficiently. Modeling and forecasting tools for hydro-environmental variables play a crucial role in water resource management by facilitating the development of strategies to address future conditions, such as floods and droughts, ensuring that the population is adequately water and energy supplied. However, existing stochastic time series models in the literature fail to incorporate asymmetric distributions with double-bounded support and also do not adequately address stochastic seasonality or exogenous regressors within the systematic component. Including these characteristics in a model would significantly expand the potential for modeling and forecasting a wide range of hydro-environmental variables.

Our goal is to propose a new dynamic time series model based on the MK distribution. This model, named the modified Kumaraswamy seasonal autoregressive moving average model with exogenous regressors (MKSARMAX), is designed to model the conditional median of seasonal double-bounded time series. The model consists of autoregressive and moving average terms, a set of regressors, a seasonal component, and a link function. Link functions are used in non-Gaussian distributed models to relate the model scale to the original data scale, allowing the use of linear models to make predictions without producing inappropriate results [8].

Being a new approach to modeling time series, the MKSARMAX model considers the time dependence in the data, specifically, that the value of today is related to past values, past error predictions, or both. The time dependence can be measured by autocorrelation, also known as serial correlation. When time dependence is not considered in the model, which is the case of linear regression models, the regression assumption of no autocorrelation of the errors is not achieved. It follows that the hypothesis significance tests and confidence intervals would be incorrect, with an increased risk of type I errors, more likely indicating that the parameters are significant when they are not [23]. Therefore, it is essential to model time series using an approach that accounts for the inherent time dependence present in this type of data.

In addition to considering a more flexible distribution in the random component compared to competitors, such as the KARMA and βSARMA models, the MKSARMAX model is the only one that accommodates both stochastic seasonality and exogenous regressors in the systematic component. For the proposed model, parameter estimation, conditional observed information matrix, validation tests, prediction, and forecast are introduced. Synthetic and observed double-bounded hydro-environmental time series were considered to numerically assess the performance of the proposed model.

The paper is organized as follows. In Sect 2, we introduce the mathematical formalism of the proposed model. The conditional maximum likelihood estimation and hypothesis testing inference are shown in Sect 3. Model validation, including diagnostic analysis, model selection, prediction, and forecasting, are discussed in Sect 4. Monte Carlo simulations and two applications to observed datasets can be found in Sect 5 to evaluate the performance of the proposed model. The conclusion is presented in Sect 6. The derivation of the observed information matrix is presented in S1 Appendix, and supplementary simulation results are included in S2 Appendix.

## 2 The proposed model

This section reviews the MK distribution, introduces the median-based reparameterization of this distribution, and derives a new dynamic model suitable for modeling the conditional median of MK-distributed time series.

### 2.1 The modified Kumaraswamy (MK) distribution

The MK distribution proposed in [2] is based on a transformation, *Y* = [1 − $\log(Z){]}^{-1}$, of a Kumaraswamy-distributed variable *Z*. The random variable *Y* assumes values *y* in the unit interval and has the probability density function and the cumulative distribution function given, respectively, by

$$f(y;\alpha ,\lambda )=\frac{\alpha \lambda {\text{e}}^{\alpha -\alpha /y}(1-{\text{e}}^{\alpha -\alpha /y}{)}^{\lambda -1}}{y^{2}},$$ (1)

$$F(y;\alpha ,\lambda )=1-(1-{\text{e}}^{\alpha -\alpha /y}{)}^{\lambda },$$

being α>0 and λ>0 the shape parameters. The quantile function, useful to generate pseudo-random variables, computes the quantile τ and is given by

$$F^{-1}(\tau ;\alpha ,\lambda )=\frac{\alpha }{\alpha -\log(1-(1-\tau {)}^{1/\lambda })}.$$ (2)

Note that when τ=0.5, we have the median (μ) of *Y*, i.e. $\mu =F^{-1}(0.5;\alpha ,\lambda )$.

### 2.2 The dynamic MKSARMAX model

Parametric statistical models typically aim to model a central tendency measure, such as mean or median. However, the original parameters of the MK distribution lack direct physical or statistical interpretations. Therefore, a median-based reparameterization for the MK distribution is considered when proposing the MK-based dynamic model. From Eq 2, the following relations can be derived:


$$\alpha =\frac{\mu \log[1-(0.5{)}^{1/\lambda }]}{\mu -1},$$

$$\lambda =\frac{\log(0.5)}{\log(1-{\text{e}}^{\alpha -\alpha /\mu })}.$$

Substituting the quantity λ described above in the Eqs 1 and (2), we obtain the median-based MK distribution denoted as MK(μ,α), as detailed below.

Let ${\left\{Y_{t}\right\}}_{t\in \mathbb{Z}}$ be a stochastic process which each *Y*_*t*_ assumes values *y*_*t*_ in the interval (0,1) with probability 1, and let ${ℱ}_{t-1}$ denote the σ-field generated by the previous observations of *y*_*t*_. Assume that each $Y_{t}|{ℱ}_{t-1}\sim \text{MK}(\mu_{t},\alpha )$. With the median-based parametrization of *Y*_*t*_, the conditional density, cumulative distribution, and quantile functions are given, respectively, by


$$f_{Y}(y_{t};\mu_{t},\alpha |{ℱ}_{t-1})=\frac{\alpha \log(0.5){\text{e}}^{\alpha -\alpha /y_{t}}(1-{\text{e}}^{\alpha -\alpha /y_{t}}{)}^{(\log(0.5)/\log(1-{\text{e}}^{\alpha -\alpha /\mu_{t}}))-1}}{\log(1-{\text{e}}^{\alpha -\alpha /\mu_{t}})y_{t}^{2}},$$

$$F_{Y}(y_{t};\mu_{t},\alpha |{ℱ}_{t-1})=1-(1-{\text{e}}^{\alpha -\alpha /y_{t}}{)}^{\log(0.5)/\log(1-{\text{e}}^{\alpha -\alpha /\mu_{t}})},$$

$$F_{Y}^{-1}(u;\mu_{t},\alpha |{ℱ}_{t-1})=\frac{\alpha }{\alpha -\log\left(1-(1-u{)}^{\log(1-{\text{e}}^{\alpha -\alpha /\mu_{t}})/\log(0.5)}\right)},$$

where $0<\mu_{t}<1$ is the conditional median and α>0 is a shape parameter.

The dynamic structure of the proposed MKSARMAX is defined according to

$$\Phi (B^{S})\phi (B)(g(Y_{t})-{𝐱}_{t}\beta )=\beta_{0}+\Theta (B^{S})\theta (B)r_{t},$$ (3)

where *B* is the backshift operator such that $B^{d}g(Y_{t})=g(Y_{t-d})$ for a nonnegative integer *d*, g(·) is a strictly monotone and twice differentiable link function such that g:(0,1)→ℝ, ${𝐱}_{t}$ is the *k*–dimensional vector containing the exogenous regressors at time *t*, $\beta =(\beta_{1},\beta_{2},\ldots ,\beta_{k}{)}^{⊤}$ is the *k*–dimensional vector of unknown parameters associated to exogenous regressors ${𝐱}_{t}$, and $\beta_{0}$ is the intercept. The autoregressive and moving average terms are defined as in [1]: (i) $\Phi (B^{S})=1-\Phi_{1}B^{S}-\Phi_{2}B^{2S}-\cdots -\Phi_{P}B^{PS}$ is the seasonal autoregressive operator, considered as a polynomial in ${\text{B}}^{\text{S}}$ of degree *P*, where *P* is the seasonal autoregressive order of the model and *S* is the seasonal frequency; (ii) $\phi (B)=1-\phi_{1}B-\phi_{2}B^{2}-\cdots -\phi_{p}B^{p}$ is the autoregressive operator, where *p* is the autoregressive order; (iii) $\Theta (B^{S})=1-\Theta_{1}B^{S}-\Theta_{2}B^{2S}-\cdots -\Theta_{Q}B^{QS}$ is the seasonal moving average operator, where *Q* is the seasonal moving average order; and (iv) $\theta (B)=1-\theta_{1}B-\theta_{2}B^{2}-\cdots -\theta_{q}B^{q}$ is the moving average operator, where *q* is the non-seasonal moving average order. The error term is considered in the predictor scale, $r_{t}=g(Y_{t})-g(\mu_{t})$, as considered in [9]. The model name is adapted according to the terms included, e.g., MKSARMA$(p,q)\times (P,Q{)}_{S}$ when it does not present exogenous regressors, and MKARMAX(*p*,*q*) when it does not present stochastic seasonality.

The general MKSARMAX$(p,q)\times (P,Q{)}_{S}$ model can also be written from (3) in the following GLM-like structure

$$\eta_{t}=g(\mu_{t})=\;\beta_{0}+{𝐱}_{t}^{⊤}\beta +\sum_{i=1}^{p}\phi_{i}[g(Y_{t-i})-{𝐱}_{t-i}^{⊤}\beta ]+\sum_{j=1}^{P}\Phi_{j}[g(Y_{t-jS})-{𝐱}_{t-jS}^{⊤}\beta ]-\;\;\sum_{i=1}^{p}\sum_{j=1}^{P}\phi_{i}\Phi_{j}[g(Y_{t-(i+jS)})-{𝐱}_{t-(i+jS)}^{⊤}\beta ]-\sum_{a=1}^{q}\theta_{a}r_{t-a}-\;\;\sum_{b=1}^{Q}\Theta_{b}r_{t-bS}+\sum_{a=1}^{q}\sum_{b=1}^{Q}\theta_{a}\Theta_{b}r_{t-(a+bS)}.$$ (4)

The dynamic model structure presented above is similar to βSARMA [21]. However, our approach diverges by employing the MK distribution (which has been shown to be a better tool for hydro-environmental data modeling) and exogenous regressors. Furthermore, it is observed that the exogenous regressors are modeled separately from the intercept, which differs from the dynamic structure considered in [24]. The inclusion of exogenous regressors in the model can be extremely powerful since external variables can impact the time series behavior and improve predictions, as demonstrated in [25] and [26]. By considering exogenous regressors, the model can accommodate level changes, deterministic trends, or any deterministic action over the time series, thereby enabling the handling of human interventions in the hydrological cycle and adequately modeling level changes in double-bounded time series data, for example.

Conditions for stationarity, causality, and invertibility for dynamic time series models that assume conditional probability structures under non-Gaussian distributions remain an open and challenging topic in the literature. This limitation primarily arises because, for link functions other than the identity, the moving average error terms do not form a martingale difference sequence. As a result, the first two moments of the marginal distribution become analytically intractable, as discussed in [5], where the authors briefly addressed this point through a simulation-based approach. Nevertheless, this theoretical characteristic does not compromise the practical applicability of such models, which are widely employed in various fields (see, e.g., [27–30]). An important point we can assert regarding stationarity in double-bounded time series is that, due to the bounded support, both mean and variance are finite by construction.

**Remark 1.** *The analysis in this paper focuses on the median of a time series with values in the interval (0,1). Therefore, appropriate link functions satisfying g:(0,1)→ℝ include the logit, probit, and cloglog functions.*

**Remark 2.** *It is pointed out that a quantile approach can be considered for the MKSARMAX$(p,q)\times (P,Q{)}_{S}$ when τ∈(0,1) in (2). While this option is available for the model, the analysis in this paper focuses on the median due to its interpretability as a measure of central tendency.*

## 3 Conditional likelihood inference

In this section, we present the theory of conditional likelihood inference for the parameters, covering point estimation, hypothesis testing inference, and confidence intervals.

### 3.1 Parameters estimation

Let $y_{1},\ldots ,y_{n}$ be a sample of a MKSARMAX(p,q) × (*P*,*Q*)_*S*_ stochastic process and $\gamma =(\beta_{0},\beta^{⊤},\phi^{⊤},\theta^{⊤},\Phi^{⊤},\Theta^{⊤},\alpha {)}^{⊤}$ be the (ω+k+2)-dimensional parameter vector, where ω=p+q+P+Q. The conditional maximum likelihood estimators (CMLE) for model parameters are obtained by maximizing the logarithm of the conditional likelihood function. To achieve this, the score vector, which consists of the derivatives of the conditional log-likelihood function with respect to the parameters, is expressed as

$$𝐔(\gamma )=\frac{\partial \ell }{\partial \gamma }={\left(\frac{\partial \ell }{\partial \beta_{0}},\frac{\partial \ell }{\partial \beta^{⊤}},\frac{\partial \ell }{\partial \phi^{⊤}},\frac{\partial \ell }{\partial \theta^{⊤}},\frac{\partial \ell }{\partial \Phi^{⊤}},\frac{\partial \ell }{\partial \Theta^{⊤}},\frac{\partial \ell }{\partial \alpha }\right)}^{⊤},$$ (5)

where ℓ is the logarithm of the conditional likelihood function of the parameter vector γ conditional on the δ=max(p+PS,q+QS) initial observations, and it is given by


$$\ell =\sum_{t=\delta +1}^{n}\log(f_{Y}(y_{t};\mu_{t},\alpha |{ℱ}_{t-1}))=\sum_{t=\delta +1}^{n}\ell_{t}(\mu_{t},\alpha ),$$

where


$$\ell_{t}(\mu_{t},\alpha )=\log(\alpha )+\alpha -\frac{\alpha }{y_{t}}+\left[\frac{\log(0.5)}{\mu_{t}^{⋆}}-1\right]y_{t}^{⋆}+\log\left(\frac{\log(0.5)}{\mu_{t}^{⋆}}\right)-2\log(y_{t}),$$

being $\mu_{t}^{⋆}=\log\left(1-{\text{e}}^{\alpha -\alpha /\mu_{t}}\right)$ and $y_{t}^{⋆}=\log\left(1-{\text{e}}^{\alpha -\alpha /y_{t}}\right)$.

Considering the chain rule to compute the derivatives of Eq 5, for $\gamma_{h}\neq \alpha$ and h=1,…,(ω+k+1), we have


$$\frac{\partial \ell }{\partial \gamma_{h}}=\sum_{t=\delta +1}^{n}\frac{\partial \ell_{t}(\mu_{t},\alpha )}{\partial \mu_{t}}\frac{\partial \mu_{t}}{\partial g(\mu_{t})}\frac{\partial g(\mu_{t})}{\partial \gamma_{h}},$$

where


$$\frac{\partial \ell_{t}(\mu_{t},\alpha )}{\partial \mu_{t}}=\frac{\alpha {\text{e}}^{\alpha }\left(\mu_{t}^{⋆}+\log(0.5)y_{t}^{⋆}\right)}{\mu_{t}^{2}\left[{\text{e}}^{\alpha /\mu_{t}}-{\text{e}}^{\alpha }\right](\mu_{t}^{⋆}{)}^{2}},$$

and


$$\frac{\partial \mu_{t}}{\partial g(\mu_{t})}=(g^{-1}{)}^{\prime }(g(\mu_{t}))=\frac{1}{g^{\prime }(\mu_{t})},$$

being the derivative of the inverse of the link function g(·). The derivatives of $g(\mu_{t})$ with respect to the parameters $\beta_{0},\beta^{⊤},\phi^{⊤},\theta^{⊤},\Phi^{⊤}$, and $\Theta^{⊤}$, considering the general form of the model, are computed as


$$\frac{\partial g(\mu_{t})}{\partial \beta_{0}}=1+\sum_{a=1}^{q}\theta_{a}\frac{\partial g(\mu_{t-a})}{\partial \beta_{0}}+\sum_{b=1}^{Q}\Theta_{b}\frac{\partial g(\mu_{t-bS})}{\partial \beta_{0}}-\;\sum_{a=1}^{q}\sum_{b=1}^{Q}\theta_{a}\Theta_{b}\frac{\partial g(\mu_{t-(a+bS)})}{\partial \beta_{0}},\;\frac{\partial g(\mu_{t})}{\partial \beta_{c}}=x_{t,c}-\sum_{i=1}^{p}\phi_{i}x_{t-i,c}-\sum_{j=1}^{P}\Phi_{j}x_{t-jS,c}+\sum_{i=1}^{p}\sum_{j=1}^{P}\phi_{i}\Phi_{j}x_{t-(i+jS),c}+\;\sum_{a=1}^{q}\theta_{a}\frac{\partial g(\mu_{t-a})}{\partial \beta_{c}}+\sum_{b=1}^{Q}\Theta_{b}\frac{\partial g(\mu_{t-bS})}{\partial \beta_{c}}-\sum_{a=1}^{q}\sum_{b=1}^{Q}\theta_{a}\Theta_{b}\frac{\partial g(\mu_{t-(a+bS)})}{\partial \beta_{c}},\;\frac{\partial g(\mu_{t})}{\partial \phi_{i}}=[g(y_{t-i})-{𝐱}_{t-i}\beta ]-\sum_{j=1}^{P}\Phi_{j}[g(y_{t-(i+jS)})-{𝐱}_{t-(i+jS)}\beta ]+\;\sum_{a=1}^{q}\theta_{a}\frac{\partial g(\mu_{t-a})}{\partial \phi_{i}}+\sum_{b=1}^{Q}\Theta_{b}\frac{\partial g(\mu_{t-bS})}{\partial \phi_{i}}-\sum_{a=1}^{q}\sum_{b=1}^{Q}\theta_{a}\Theta_{b}\frac{\partial g(\mu_{t-(a+bS)})}{\partial \phi_{i}},\;\frac{\partial g(\mu_{t})}{\partial \theta_{a}}=-r_{t-a}+\sum_{b=1}^{Q}\Theta_{b}r_{t-(a+bS)}+\sum_{a=1}^{q}\theta_{a}\frac{\partial g(\mu_{t-a})}{\partial \theta_{a}}+\sum_{b=1}^{Q}\Theta_{b}\frac{\partial g(\mu_{t-bS})}{\partial \theta_{a}}-\;\sum_{a=1}^{q}\sum_{b=1}^{Q}\theta_{a}\Theta_{b}\frac{\partial g(\mu_{t-(a+bS)})}{\partial \theta_{a}},\;\frac{\partial g(\mu_{t})}{\partial \Phi_{j}}=[g(y_{t-jS})-{𝐱}_{t-jS}\beta ]-\sum_{i=1}^{p}\phi_{i}[g(y_{t-(i+jS)})-{𝐱}_{t-(i+jS)}\beta ]+\;\sum_{a=1}^{q}\theta_{a}\frac{\partial g(\mu_{t-a})}{\partial \Phi_{j}}+\sum_{b=1}^{Q}\Theta_{b}\frac{\partial g(\mu_{t-bS})}{\partial \Phi_{j}}-\sum_{a=1}^{q}\sum_{b=1}^{Q}\theta_{a}\Theta_{b}\frac{\partial g(\mu_{t-(a+bS)})}{\partial \Phi_{j}},\;\frac{\partial g(\mu_{t})}{\partial \Theta_{b}}=-r_{t-bS}+\sum_{a=1}^{q}\theta_{a}r_{t-(a+bS)}+\sum_{a=1}^{q}\theta_{a}\frac{\partial g(\mu_{t-a})}{\partial \Theta_{b}}+\sum_{b=1}^{Q}\Theta_{b}\frac{\partial g(\mu_{t-bS})}{\partial \Theta_{b}}-\;\sum_{a=1}^{q}\sum_{b=1}^{Q}\theta_{a}\Theta_{b}\frac{\partial g(\mu_{t-(a+bS)})}{\partial \Theta_{b}}.$$

Finally, the derivative of $\ell_{t}(\mu_{t},\alpha )$ with respect to the parameter α is given by


$$\frac{\partial \ell_{t}(\mu_{t},\alpha )}{\partial \alpha }=1+\frac{1}{\alpha }+\frac{\varphi_{t}\log(0.5)y_{t}^{⋆}}{\mu_{t}^{⋆}}-\frac{(y_{t}-1)\left[\log(0.5)/\mu_{t}^{⋆}-1\right]{\text{e}}^{\alpha -\alpha /y_{t}}}{y_{t}-y_{t}{\text{e}}^{\alpha -\alpha /y_{t}}}+\varphi_{t}-\frac{1}{y_{t}},$$

where


$$\varphi_{t}=\frac{(\mu_{t}-1){\text{e}}^{\alpha }}{\mu_{t}\left({\text{e}}^{\alpha /\mu_{t}}-{\text{e}}^{\alpha }\right)\mu_{t}^{⋆}}.$$

The CMLE of γ, $\hat{\gamma }=({\hat{\beta }}_{0},{\hat{\beta }}^{⊤},{\hat{\phi }}^{⊤},{\hat{\theta }}^{⊤},{\hat{\Phi }}^{⊤},{\hat{\Theta }}^{⊤},\hat{\alpha }{)}^{⊤}$, is obtained by solving

𝐔(γ)=0, (6)

where 0 is the (ω+k+2)-dimensional vector of zeros, whose solution has no closed-form. The Broyden-Fletcher-Goldfarb-Shanno (BFGS) method [31] with analytic first derivatives was chosen as the nonlinear optimization algorithm to solve the system in Eq 6. The initial values for the iterative method were set as follows: (i) θ and Θ were set as zero as in [21]; (ii) α was obtained using the generalized simulated annealing function [32] as in [2]; (iii) $\beta_{0}$, β, ϕ, and Φ were derived from the ordinary least squares estimate associated with the linear regression where the response vector is given by $g(y_{\delta +1}),g(y_{\delta +2}),\ldots ,g(y_{n})$, and the matrix of the independent variables is given by


$$\left[\begin{array}{ccccccc} 1 & x_{\delta +1,1} & \ldots & x_{\delta +1,k} & g(y_{\delta +1-i}) & \ldots & g(y_{\delta +1-jS})\\ 1 & x_{\delta +2,1} & \ldots & x_{\delta +2,k} & g(y_{\delta +2-i}) & \ldots & g(y_{\delta +2-jS})\\ \vdots & \vdots & \ddots & \vdots & \vdots & \ddots & \vdots \\ 1 & x_{n,1} & \ldots & x_{n,k} & g(y_{n-i}) & \ldots & g(y_{n-jS}) \end{array}\right],$$

similar to [21] and [33].

### 3.2 Hypothesis testing inference and confidence intervals

Based on the Central Limit Theorem, the CMLE follows normal distribution asymptotically, with the respective parameters as the mean and the inverse of the observed information matrix ( **K**) as the covariance matrix. The asymptotic variances of the estimators ${\hat{\beta }}_{0}$, $\hat{\beta }$, $\hat{\phi }$, $\hat{\theta }$, $\hat{\Phi }$, $\hat{\Theta }$, and $\hat{\alpha }$—useful for hypothesis testing inference—are the diagonal elements of the variance-covariance matrix of CMLE, which is obtained by the inverse of **K**, ${𝐊}^{-1}$. The closed-form expression for **K**, whose elements are the negative second derivatives of the conditional log-likelihood function, is derived in detail in S1 Appendix.

The test for


$${ℋ}_{0}:\;\;\gamma_{m}=0\;\text{(null parameter),}$$

$${ℋ}_{1}:\;\;\gamma_{m}\neq 0\;\text{(significant parameter),}$$

where $\gamma_{m}$, for m=1,…,(ω+k+2), represents *m*th component of γ, can be performed based on the signed square root of Wald’s statistic [34,35], which is given by


$$Z_{m}=\frac{{\hat{\gamma }}_{m}-0}{\sqrt{\mathrm{Var}({\hat{\gamma }}_{m})}},$$

where $\mathrm{Var}({\hat{\gamma }}_{m})$ is the estimated asymptotic variance of ${\hat{\gamma }}_{m}$, which is given from the diagonal of ${𝐊}^{-1}$ evaluated at the (ω  +  *k*  +  2)-dimensional CMLE vector $\hat{\gamma }$. When |*Z*_*m*_| is greater than the $z_{\left(1-\kappa /2\right)}$, the null hypothesis is rejected considering a significance level of κ∈(0,1), being $z_{\left(1-\kappa /2\right)}$ the (1−κ/2)th quantile of the standard normal distribution.

Confidence intervals for $\gamma_{m}$, with confidence approximately 100(1 − κ)%, can be derived based on the asymptotic normal distribution of the CMLE according to


$$\left[{\hat{\gamma }}_{m}-z_{\left(1-\kappa /2\right)}\sqrt{\mathrm{Var}({\hat{\gamma }}_{m})};{\hat{\gamma }}_{m}+z_{\left(1-\kappa /2\right)}\sqrt{\mathrm{Var}({\hat{\gamma }}_{m})}\right].$$

## 4 Validation, model selection, prediction and forecasting

This section presents the residual diagnostic analysis, model selection, prediction, and forecasting for the MKSARMAX model. For that, it is necessary to compute the fitted values ${\hat{\mu }}_{t}$, t=δ  +  1,…,n. The estimated ${\hat{\mu }}_{t}$ is obtained by replacing the model parameters with their estimators $\hat{\beta_{0}}$, $\hat{\beta }$, $\hat{\phi }$, $\hat{\theta }$, $\hat{\Phi }$, $\hat{\Theta }$, and $\hat{\alpha }$ in Eq 4, and applying the inverse link function to ${\hat{\eta }}_{t}$ as defined in Sect 4.3.

### 4.1 Residuals and goodness-of-fit tests

After the parameters have been estimated, diagnostic checks and goodness-of-fit tests of the fitted model should be considered. For that, the quantile residual can be performed. This residual is widely employed in the literature since it is approximately Gaussian distributed with a zero mean and unit variance when the model is correctly fitted [36], and is given by


$${\hat{e}}_{t}^{q}=F_{N(0,1)}^{-1}(F_{Y}(y_{t};{\hat{\mu }}_{t},\hat{\alpha }|{ℱ}_{t-1})),$$

where $F_{N(0,1)}^{-1}$ denotes the standard normal quantile function.

After fitting the MKSARMAX model, the residuals are expected to be approximately normally distributed, uncorrelated, exhibit a mean close to zero, and display constant variance. The presence of these properties suggests that the model has adequately captured the underlying structure of the time series and that no significant patterns remain unexplained.

To verify the goodness-of-fit of the adjusted model, the Ljung-Box test [37], Jarque–Bera test [38], and autoregressive conditional heteroskedasticity (ARCH) test [39] can be considered over the residual series to assess non-autocorrelation, Gaussianity, and non-heteroscedasticity, respectively.

### 4.2 Model selection criteria

To select the order of the MKSARMAX model for a given time series, the modified Bayesian information criterion (MBIC) proposed by [21] can be applied. The MBIC is given by


$$\text{MBIC}=-2\hat{\ell }\times \frac{n}{n-\delta }+\log(n)(\omega +k+2),$$

where $\hat{\ell }$ is the maximized conditional log-likelihood function of the fitted model. The MBIC is advantageous over the Bayesian information criterion (BIC) [40,41] in conditional likelihood inference, when the δ initial observations are not fitted, because it avoids penalizing models erroneously with numerous parameters. The BIC is chosen over AIC due to its more parsimonious overfit penalizing. The selected model has the lowest MBIC value among a set of competing fitted models.

### 4.3 Prediction and forecasting

In-sample predictions for $y_{\delta +1},\ldots ,y_{n}$ are obtained from the fitted values ${\hat{\mu }}_{\delta +1},\ldots ,{\hat{\mu }}_{n}$ as seen previously. Out-of-sample predictions, i.e., forecasting values *H* steps ahead for $y_{n+1},\ldots ,y_{n+H}$, are obtained by setting *r*_*t*_ = 0, for t=n+1,…,n+H, besides replacing the model parameters with their estimates in Eq 4, and applying the inverse of the link function to ${\hat{\eta }}_{t}$ for the rolling window forecast and by also setting *g*(*y*_*t*_) as $g({\hat{\mu }}_{t})$ for the traditional forecast. So the general form of prediction and forecasting is given by


$${\hat{\mu }}_{t}=g^{-1}({\hat{\beta }}_{0}+{𝐱}_{t}^{⊤}\hat{\beta }+\sum_{i=1}^{p}{\hat{\phi }}_{i}[g^{*}(y_{t-i})-{𝐱}_{t-i}^{⊤}\hat{\beta }]+\sum_{j=1}^{P}{\hat{\Phi }}_{j}[g^{*}(y_{t-jS})-{𝐱}_{t-jS}^{⊤}\hat{\beta }]-\mathrm{\backslash nonumber}\;\sum_{i=1}^{p}\sum_{j=1}^{P}{\hat{\phi }}_{i}{\hat{\Phi }}_{j}[g^{*}(y_{t-(i+jS)})-{𝐱}_{t-(i+jS)}^{⊤}\hat{\beta }]-\sum_{a=1}^{q}{\hat{\theta }}_{a}r_{t-a}^{*}-\sum_{b=1}^{Q}{\hat{\Theta }}_{b}r_{t-bS}^{*}+\mathrm{\backslash nonumber}\;\sum_{a=1}^{q}\sum_{b=1}^{Q}{\hat{\theta }}_{a}{\hat{\Theta }}_{b}r_{t-(a+bS)}^{*}),$$

where


$$g^{*}(y_{t})=\left\{ \begin{array}{c} g(y_{t}),\;\;\text{if}\;\;1\leq t\leq n\\ g({\hat{\mu }}_{t}),\;\;\text{if}\;\;t>n\;\;\text{for traditional forecasting}\\ g(y_{t}),\;\;\text{if}\;\;t>n\;\;\text{for rolling window forecasting} \end{array}\right.,$$

$$r_{t}^{*}=\left\{ \begin{array}{c} 0,\;\;\text{if}\;\;t\leq \delta \\ g(y_{t})-g({\hat{\mu }}_{t}),\;\;\text{if}\;\;(\delta +1)\leq t\leq n\\ 0,\;\;\text{if}\;\;t>n \end{array}\right..$$

## 5 Numerical evaluation and discussion

In this section, Monte Carlo simulations are presented and discussed to assess the introduced model using synthetic data. Different sample sizes and model structures are employed for this purpose. Additionally, applications to useful water volume datasets are performed by comparing the proposed and some competing models. The considered link function is the logit, i.e., $g(\mu )=\log(\frac{\mu }{1-\mu })$, which is the most common choice to other models for double-bounded data in the interval (0,1), such as KARMA and βARMA models [3,9,11,13,27]. The significance level for hypothesis testing is set at 5% for Wald test, Ljung-Box test, Jarque-Bera test, and ARCH test, and is set at 10% for Diebold-Mariano test. Implementations in the R language [42] for MKSARMAX model fitting are available [43].

### 5.1 Monte Carlo simulations

Monte Carlo simulations were performed to evaluate the finite sample performance of the CMLE on synthetic hydro-environmental data. The parameters used for generating synthetic hydro-environmental time series were obtained by fitting real datasets with the MKSARMAX model, enabling their evaluation in the simulation study. We simulated 5000 replications of synthetic hydro-environmental time series considering four different sample sizes, n∈{100,300,500,700}. The mean, average bias, relative bias, standard error (SE), and mean square error (MSE) were adopted as figures of merit to numerically evaluate the point estimators of the model parameters. The coverage rates (CR) of the confidence intervals were computed to evaluate interval estimation. In each Monte Carlo replication, the inversion method was employed to simulate MK distributed hydro-environmental time series with $\mu_{t}$ based on Eq 4.

The simulation results of MKSARMA (1,1) × (1,1)_12_ scenario are presented in Table 1; the parameter values are presented inside the parentheses. For this scenario, one model was discarded in the simulations for n=(100,300,500), and two models were discarded in the simulations for n=(700), due to failure to converge using the BFGS optimization method or a non–positive-definite observed information matrix. As expected, bias and MSE figures reduce as *n* grows. This behavior evidences the consistency of the CMLE. The coverage rate is close to the nominal value, 95%, especially for the sample sizes equal to 500 and 700. Note that: (i) ${\hat{\phi }}_{1}$, ${\hat{\theta }}_{1}$, and $\hat{\alpha }$ excel in terms of relative bias; (ii) ${\hat{\theta }}_{1}$ relative bias converges faster to zero than the other estimators; (iii) the seasonal parameter estimators, ${\hat{\Phi }}_{1}$ and ${\hat{\Theta }}_{1}$, display the highest relative bias values—such fact was also discussed in [24] regarding the CMP-ARMA$(1,1)\times (1,1{)}_{12}$ model, where it was found, by simulation studies, that inferences about seasonal parameters estimators are more biased; and (iv) $\hat{\alpha }$ shows the highest MSE values.

**Table 1:** Mean, bias, relative bias, standard error, and MSE of MKSARMA $(1,1)\times (1,1{)}_{12}$ parameter estimators, and coverage rate for the confidence interval. Parameter values are presented inside the parentheses.

Measures	$\beta_{0}$	$\phi_{1}$	$\theta_{1}$	$\Phi_{1}$	$\Theta_{1}$	α
(0.5290)	(0.4229)	(−0.3380)	(0.2971)	(−0.3747)	(14.9843)
Sample size = 100						
Mean	0.4843	0.4145	–0.3473	0.3795	–0.3069	15.5535
Bias	–0.0447	–0.0084	–0.0093	0.0824	0.0678	0.5692
Bias (%)	8.4460	1.9981	2.7620	27.7455	18.0926	3.7990
SE	0.1409	0.1284	0.1232	0.1357	0.1586	1.4669
MSE	0.0219	0.0166	0.0153	0.0252	0.0298	2.4759
CR	0.8670	0.9316	0.9142	0.8588	0.8960	0.9290
Sample size = 300						
Mean	0.5181	0.4199	–0.3411	0.3176	–0.3589	15.1034
Bias	–0.0109	–0.0030	–0.0031	0.0205	0.0158	0.1191
Bias (%)	2.0678	0.7091	0.9261	6.9061	4.2296	0.7945
SE	0.0685	0.0617	0.0571	0.0671	0.0640	0.7578
MSE	0.0048	0.0038	0.0033	0.0049	0.0043	0.5884
CR	0.9292	0.9468	0.9344	0.9242	0.9384	0.9430
Sample size = 500						
Mean	0.5237	0.4204	–0.3394	0.3084	–0.3664	15.0577
Bias	–0.0053	–0.0025	–0.0014	0.0113	0.0083	0.0734
Bias (%)	1.0036	0.5936	0.4250	3.7917	2.2281	0.4899
SE	0.0532	0.0461	0.0421	0.0511	0.0463	0.5879
MSE	0.0029	0.0021	0.0018	0.0027	0.0022	0.3510
CR	0.9354	0.9456	0.9412	0.9384	0.9398	0.9408
Sample size = 700						
Mean	0.5265	0.4219	–0.3382	0.3030	–0.3705	15.0405
Bias	–0.0025	–0.0010	–0.0002	0.0059	0.0042	0.0562
Bias (%)	0.4672	0.2386	0.0494	1.9854	1.1196	0.3747
SE	0.0462	0.0395	0.0347	0.0444	0.0387	0.5007
MSE	0.0021	0.0016	0.0012	0.0020	0.0015	0.2539
CR	0.9400	0.9386	0.9402	0.9320	0.9442	0.9436

Simulation results of MKARMAX (1,1) scenario are presented in Table 2. For this scenario, one exogenous regressor **x** is changing the level of the time series in the middle of the sample size, being $x_{1},\ldots ,x_{n/2}=0$ and $x_{n/2+1},\ldots ,x_{n}=1$. As expected, bias and MSE reduce as *n* increases, and the coverage rate is close to the nominal value, 95%, for the sample sizes n=(300,500,700). Note that: (i) the moving average parameter estimators, ${\hat{\theta }}_{1}$, display the highest average relative bias values; (ii) $\hat{\alpha }$ shows the highest MSE values; and (iii) ${\hat{\beta }}_{1}$ and $\hat{\alpha }$ excel in terms of relative bias. Complementary simulation results for MKSARMA (1,0) × (1,0)_12_ and MKSARMA (0,1) × (0,1)_12_ are presented in S2 Appendix. All the models successfully converged and the observed information matrices were positive definite in the MKARMAX (1,1), MKSARMA (1,0) × (1,0)_12_, and MKSARMA (0,1) × (0,1)_12_ scenarios, ensuring the reliability of the estimated parameters.

**Table 2:** Mean, bias, relative bias, standard error, and MSE of MKARMAX (1,1) parameter estimators, and coverage rate for the confidence interval. Parameter values are presented inside the parentheses.

Measures	$\beta_{0}$	$\beta_{1}$	$\phi_{1}$	$\theta_{1}$	α
(0.6828)	(−0.1031)	(0.5152)	(−0.1389)	(14.3650)
Sample size = 100					
Mean	0.7383	–0.1050	0.4760	–0.1702	14.8830
Bias	0.0555	–0.0019	–0.0392	–0.0313	0.5180
Bias (%)	8.1320	1.8616	7.6081	22.5452	3.6062
Standard error	0.1803	0.1161	0.1335	0.1366	1.3029
MSE	0.0356	0.0135	0.0194	0.0197	1.9659
Coverage rate	0.9412	0.9360	0.9422	0.9176	0.9372
Sample size = 300					
Mean	0.7015	–0.1044	0.5018	–0.1506	14.5044
Bias	0.0187	–0.0013	–0.0134	–0.0117	0.1394
Bias (%)	2.7329	1.2239	2.5985	8.4203	0.9707
Standard error	0.0949	0.0675	0.0701	0.0724	0.7045
MSE	0.0093	0.0046	0.0051	0.0054	0.5157
Coverage rate	0.9504	0.9458	0.9498	0.9426	0.9500
Sample size = 500					
Mean	0.6947	–0.1043	0.5069	–0.1460	14.4650
Bias	0.0119	–0.0012	–0.0083	–0.0071	0.1000
Bias (%)	1.7384	1.1828	1.6129	5.0824	0.6963
Standard error	0.0722	0.0526	0.0531	0.0554	0.5550
MSE	0.0053	0.0028	0.0029	0.0031	0.3181
Coverage rate	0.9504	0.9456	0.9472	0.9472	0.9454
Sample size = 700					
Mean	0.6898	–0.1025	0.5103	–0.1427	14.4454
Bias	0.0070	0.0006	–0.0049	–0.0038	0.0804
Bias (%)	1.0271	0.6289	0.9543	2.7601	0.5598
Standard error	0.0597	0.0447	0.0440	0.0462	0.4581
MSE	0.0036	0.0020	0.0020	0.0022	0.2164
Coverage rate	0.9520	0.9452	0.9530	0.9480	0.9454

In general, the Monte Carlo simulation results show that the performance of the conditional likelihood inference in the MKSARMAX is good for finite samples. The CMLE presents minor errors and biases as the sample size of synthetic hydro-environmental time series increases, and the coverage rate reaches close to the 95% confidence level in all analyzed scenarios. To complement the numerical evaluation of the model, the MKSARMAX fit and forecast for two real datasets of monthly useful water volume (UV) [44] are presented in the following.

### 5.2 Application to Caconde useful water volume dataset

**Fig 2:**
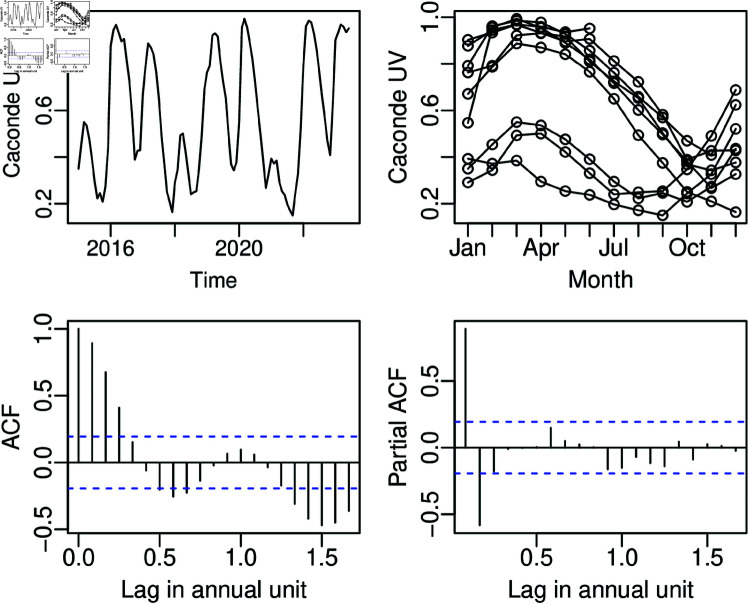
Time series, seasonal plot, ACF, and PACF for the Caconde UV dataset from t=1 to 102.

This section evaluates the effectiveness of the proposed model in the monthly useful water volume of the Caconde Reservoir, from a hydroelectric power plant situated in Caconde, SP, Brazil. This volume is defined as the percentage of a reservoir’s volume between its maximum and minimum operational levels [45]. In the case of reservoirs from hydroelectric power plant, such as the Caconde Reservoir, the useful volume represents the volume of water (in ${\text{m}}^{3}$) in the reservoir that can be effectively used for power generation and the useful volume is obtained from the ratio between the current level of the reservoir and the maximum and minimum operational levels difference [13]. The Caconde UV dataset employed in this section is from January 2015 to February 2024. The last 8 observations were used to assess the traditional and rolling window forecasting (Sect 4.3) performance of the proposed model; thus, *n* = 102 and *H* = 8. The time series is presented in Fig 2 through its time plot, seasonal plot, autocorrelation function (ACF) plot, and partial autocorrelation function (PACF) plot. The fit dataset varies from 0.1500 to 0.9930, with an unconditional median of 0.5285, a sample average of 0.5804, and a standard deviation of 0.2666. The nonparametric Friedman test (p-value<0.0001) [46] indicates a significant seasonality in the time series, which can also be seen in the Fig 2. Typically, at the end of the rainfall period, from December to April, the reservoirs reach higher useful water volume levels than in the other periods of the year.

In order to select the best MKSARMAX fitted model, only models in which (i) the BFGS method converges and (ii) the observed information matrix is positive semidefinite were considered. Some other critical points were verified, such as (iii) significant parameter coefficients (Wald test Sect 3.2); (iv) autoregressive and moving-average coefficient roots being outside the unit circle to approximately ensure the stability of *g*(*y*); and (v) quantile residuals behaving as a white noise process (Sect 4.1). If one parameter coefficient of the fitted model does not reject the null hypothesis of the Wald test, $\gamma_{m}=0$, the adjusted model is inadequate and the fit must be discarded [47]. Thus, different lags up to the defined order are considered to find the best model that fits the dataset.

It is important to check the historical facts that affect the time series to incorporate them into the model as exogenous regressors. If cycles outside of the seasonal period are identified, they can be incorporated into the model as exogenous regressors. Also, droughts or floods are critical periods in UV reservoirs, so the time series behaves differently in those periods, presenting too low UV values in droughts and too high values in floods. Between January 2015 and February 2024, we had one big water crisis in Brazil during 2021 [48]. This crisis was caused mainly by a drought period that led to low useful water volume in the reservoirs and compromised the power generation of the Caconde Reservoir, since the hydroeletric power plant had to lower the minimum flow level [49]. Consequently, energy production was reduced to extend the retention of water within the reservoirs for a prolonged period.

**Fig 3:**
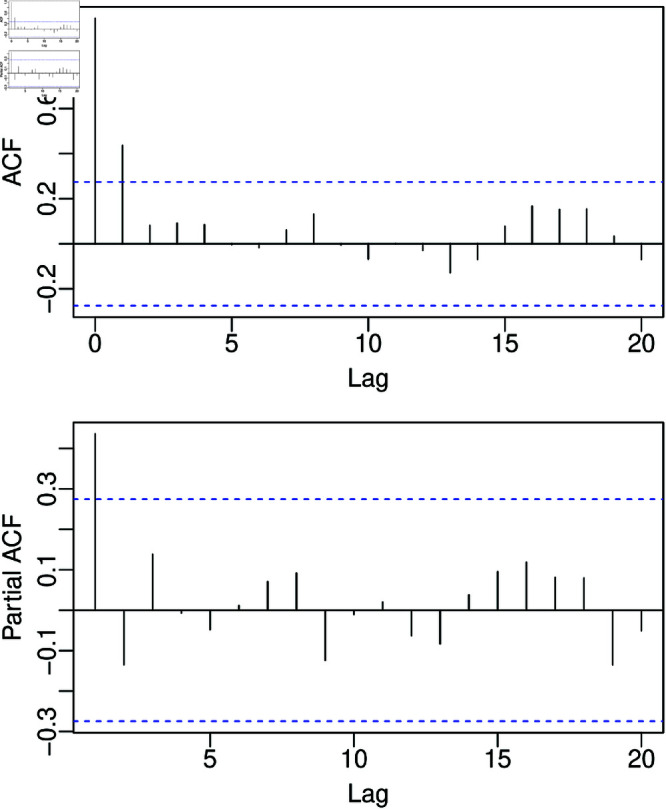
ACF and PACF of MKSARMAX $(3,2)\times (4,2{)}_{12}+X_{21}\beta_{1}$ quantile residuals for the Caconde UV dataset.

Restricting the maximum model orders *p*,*q*,*P*, and *Q* to 4 for computational simplification and considering the model selection criteria described in Sect 4.2, we successfully modeled the Caconde UV dataset using a MKSARMAX $(3,2)\times (4,2{)}_{12}+X_{21}\beta_{1}$, with $\phi_{1}=\theta_{1}=\Phi_{1}=\Phi_{2}=\Theta_{1}=0$ and *X*_21_ being an exogenous regressor changing the level of the time series in the Brazil water crisis [48] from January 2021 to December 2021. The fitted MKSARMAX $(3,2)\times (4,2{)}_{12}+X_{21}\beta_{1}$ and its diagnostic test results are presented in Table 3. According to the employed validation tests, the model residuals are independent, homoscedastic, and normally distributed. The Ljung-Box test analyzed 24 lags, and the ARCH test analyzed 10 lags. Fig 3 presents the ACF and PACF of the residuals. Those are in accordance with the Ljung-Box test evaluation of non-autocorrelation, with most autocorrelation and partial autocorrelation within the 95% confidence interval.

**Table 3:** Fitted MKSARMAX $(3,2)\times (4,2{)}_{12}+X_{21}\beta_{1}$ for the Caconde UV dataset. CMLE coefficients, confidence intervals, statistic and p-value of Wald test, and MBIC are presented, as well as the statistics and p-values of Ljung-Box, Jarque-Bera, and ARCH tests.

Parameter	CMLE	Lower bound	Upper bound	Statistic	p-value
$\beta_{0}$	0.9308	0.7199	1.1416	8.6526	<0.0001
$\beta_{1}$	–0.6282	–0.8161	–0.4403	–6.5531	<0.0001
$\phi_{2}$	–0.4107	–0.4783	–0.3432	–11.9127	<0.0001
$\phi_{3}$	–0.0519	–0.0941	–0.0098	–2.4144	0.0158
$\theta_{2}$	–0.7406	–0.7909	–0.6903	–28.8314	<0.0001
$\Phi_{3}$	0.9299	0.8517	1.0081	23.2996	<0.0001
$\Phi_{4}$	0.0969	0.0039	0.1898	2.0431	0.0410
$\Theta_{2}$	–0.7146	–0.9219	–0.5074	–6.7575	<0.0001
α	4.9861	3.5609	6.4112	6.8571	<0.0001
MBIC=−260.3796;					
Ljung-Box test statistic = 23.8335 (p-value =0.1606);					
Jarque-Bera test statistic = 1.8817 (p-value =0.3903);					
ARCH test statistic = 8.6060 (p-value =0.5699).					

**Fig 4:**
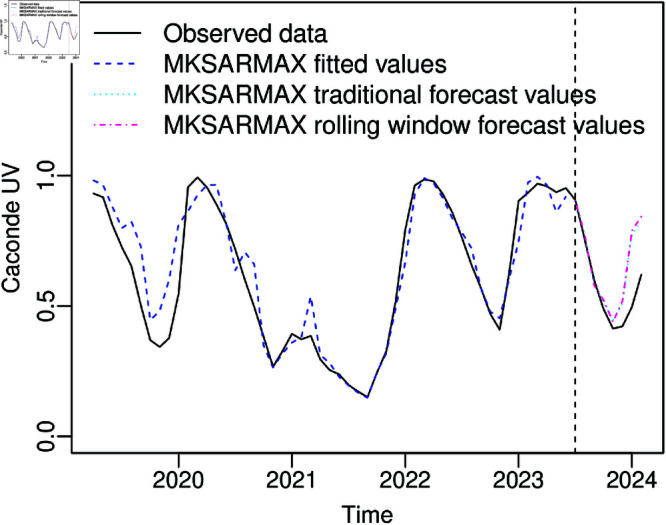
Fitted MKSARMAX $(3,2)\times (4,2{)}_{12}+X_{21}\beta_{1}$ and 8 steps traditional and rolling window forecasts for the Caconde UV dataset.

The autoregressive parameters in the model suggest that the current value is a function of past values. So, significant $\phi_{2}$ and $\phi_{3}$ inform that the UV in the second and third previous months significantly help explain the UV in the current month, and significant $\Phi_{3}$ and $\Phi_{4}$ inform that the UV in the same month three and four years ago help explain the UV in the current month. The moving average terms in the model suggest that the current value is a function of past prediction errors. So, significant $\theta_{2}$ informs that the UV error prediction in the second previous month significantly helps explain the UV in the current month, and significant $\Theta_{2}$ informs that the UV error prediction of the same month two years ago helps explain the UV in the current month. Also, the significant $\beta_{1}$ parameter and negative value of its estimator corroborate the evidence of lower useful water volume levels in the Caconde Reservoir in the year 2021. Fig 4 presents the observed and predicted values obtained considering the MKSARMAX model, displaying close predicted and observed values, corroborating the goodness-of-fit test results.

The prediction and forecast performance of the proposed MKSARMAX model is compared with the additive Holt-Winters method from the HoltWinters function of the stats base package [42], βSARMA, SARMAX, and KARMA models. We fitted the SARMAX and the βSARMA models with the same autoregressive and moving-average terms as MKSARMAX, and for the KARMA model, the exogenous regressors $\sin\left(\frac{2\pi t}{12}\right)$ and $\cos\left(\frac{2\pi t}{12}\right)$ were added to model the seasonality deterministically, once the KARMA model does not consider stochastic seasonality terms. The Diebold-Mariano test from the dm.test function of the forecast package [50] considering the absolute value loss function indicates that the MKSARMAX model has a better traditional forecast than the Holt-Winters (p-value = 0.0068) and KARMA models (p-value = 0.0009). The predicted in- and out-of-sample accuracy of the proposed model and its competitors are displayed in Table 4 and Table 5, respectively. Prediction accuracy measures, including mean average error (MAE), root mean square error (RMSE), mean absolute percentage error (MAPE), and mean directional accuracy (MDA) were considered to evaluate the models. Lower values for MAE, RMSE, and MAPE are desirable, indicating better performance. Additionally, the higher the MDA value, the better the prediction follows the time series directional movement (upward or downward).

**Table 4:** In-sample prediction accuracy measures MAE, RMSE, MAPE, and MDA for MKSARMAX, βSARMA, SARMAX, additive Holt-Winters, and KARMA models fit of observations from t=52 to 102 of the Caconde UV dataset. The best value for each measure is shaded light gray.

Model	MAE	RMSE	MAPE	MDA
MKSARMAX$(3,2)\times (4,2{)}_{12}+X_{21}\beta_{1}$	lightgray0.0584	0.0874	lightgray10.6447	0.8431
βSARMA$(3,2)\times (4,2{)}_{12}$	0.0702	0.0958	16.1703	0.8039
SARMAX$(3,2)\times (4,2{)}_{12}+X_{21}\beta_{1}$	0.0599	lightgray0.0753	11.9435	lightgray0.8627
Additive Holt-Winters	0.0759	0.1000	18.0223	0.8431
KARMA$(3,2)+X_{21}\beta_{1}+\sin\frac{2\pi t}{12}\beta_{2}+\cos\frac{2\pi t}{12}\beta_{3}$	0.0924	0.1213	19.5689	0.7255

**Table 5:** Out-of-sample traditional and rolling window forecasts accuracy measures MAE, RMSE, MAPE, and MDA for MKSARMAX, βSARMA, SARMAX, additive Holt-Winters, and KARMA models forecast of the Caconde UV dataset. The best value for each measure is shaded light gray.

Model	Traditional forecast				Rolling window forecast			
	MAE	RMSE	MAPE	MDA	MAE	RMSE	MAPE	MDA
1 step								
MKSARMAX	lightgray0.0016	lightgray0.0016	lightgray0.1767	lightgray1.0000	lightgray0.0016	lightgray0.0016	lightgray0.1767	lightgray1.0000
βSARMA	0.1378	0.1378	15.1952	lightgray1.0000	0.1378	0.1378	15.1952	lightgray1.0000
SARMAX	0.0966	0.0966	10.6545	lightgray1.0000	0.0966	0.0966	10.6545	lightgray1.0000
Holt-Winters	0.0994	0.0994	10.9617	lightgray1.0000	0.0994	0.0994	10.9617	lightgray1.0000
KARMA	0.1636	0.1636	18.0400	lightgray1.0000	0.1636	0.1636	18.0400	lightgray1.0000
2 steps								
MKSARMAX	lightgray0.0089	lightgray0.0116	lightgray1.1641	lightgray1.0000	lightgray0.0089	lightgray0.0116	lightgray1.1641	lightgray1.0000
βSARMA	0.1529	0.1537	18.6972	lightgray1.0000	0.1529	0.1537	18.6972	lightgray1.0000
SARMAX	0.0928	0.0929	11.2023	lightgray1.0000	0.0928	0.0929	11.2023	lightgray1.0000
Holt-Winters	0.0931	0.0933	11.2083	lightgray1.0000	0.0519	0.0704	5.7656	lightgray1.0000
KARMA	0.1132	0.1239	13.1632	lightgray1.0000	0.1132	0.1239	13.1632	lightgray1.0000
3 steps								
MKSARMAX	lightgray0.0122	lightgray0.0143	lightgray1.8153	lightgray1.0000	lightgray0.0128	lightgray0.0152	lightgray1.9201	lightgray1.0000
βSARMA	0.1705	0.1728	23.8917	lightgray1.0000	0.1542	0.1547	21.1714	lightgray1.0000
SARMAX	0.0723	0.0780	9.2084	lightgray1.0000	0.0833	0.0844	11.0379	lightgray1.0000
Holt-Winters	0.0647	0.0763	7.9095	lightgray1.0000	0.0616	0.0742	8.3541	lightgray1.0000
KARMA	0.1727	0.1966	24.9906	lightgray1.0000	0.1043	0.1128	13.5849	lightgray1.0000
4 steps								
MKSARMAX	lightgray0.0155	lightgray0.0178	lightgray2.6717	lightgray1.0000	lightgray0.0183	lightgray0.0218	lightgray3.2141	lightgray1.0000
βSARMA	0.1774	0.1794	28.0612	lightgray1.0000	0.1644	0.1657	25.8653	lightgray1.0000
SARMAX	0.0635	0.0700	8.8107	lightgray1.0000	0.0827	0.0836	12.4322	lightgray1.0000
Holt-Winters	0.0631	0.0722	8.9193	lightgray1.0000	0.0650	0.0743	10.1044	lightgray1.0000
KARMA	0.2032	0.2252	33.8428	0.7500	0.0852	0.0987	11.6146	lightgray1.0000
5 steps								
MKSARMAX	lightgray0.0190	lightgray0.0216	lightgray3.7199	lightgray1.0000	lightgray0.0198	lightgray0.0227	lightgray3.8304	lightgray1.0000
βSARMA	0.1871	0.1896	33.3952	lightgray1.0000	0.1748	0.1770	31.1746	lightgray1.0000
SARMAX	0.0641	0.0693	10.2686	lightgray1.0000	0.0841	0.0848	14.2887	lightgray1.0000
Holt-Winters	0.0820	0.0957	14.7811	0.8000	0.0732	0.0817	13.2254	0.8000
KARMA	0.2121	0.2298	39.0564	0.8000	0.0956	0.1075	15.9320	0.8000
6 steps								
MKSARMAX	lightgray0.0303	lightgray0.0407	lightgray6.5414	lightgray1.0000	lightgray0.0326	lightgray0.0446	lightgray7.0085	lightgray1.0000
βSARMA	0.1856	0.1878	34.8720	0.8333	0.1736	0.1755	32.5997	0.8333
SARMAX	0.0569	0.0639	9.3785	0.8333	0.0764	0.0790	13.4030	0.8333
Holt-Winters	0.1171	0.1479	23.8568	0.8333	0.0847	0.0945	16.6311	0.8333
KARMA	0.2642	0.2998	53.2707	0.8333	0.1095	0.1224	20.3515	0.8333
7 steps								
MKSARMAX	lightgray0.0663	0.1130	lightgray13.7272	lightgray1.0000	lightgray0.0697	0.1179	lightgray14.4217	lightgray1.0000
βSARMA	0.1647	0.1745	31.0040	0.8571	0.1581	0.1643	29.8152	0.8571
SARMAX	0.0776	lightgray0.0964	13.8421	0.8571	0.0903	lightgray0.0982	16.4883	0.8571
Holt-Winters	0.1752	0.2407	35.5395	0.8571	0.1069	0.1261	21.1806	0.8571
KARMA	0.2795	0.3110	56.3548	0.7143	0.1120	0.1231	21.1093	0.8571
8 steps								
MKSARMAX	0.0833	0.1277	16.0939	lightgray1.0000	0.0889	0.1357	17.1250	lightgray1.0000
βSARMA	0.1483	0.1637	27.8137	0.8750	0.1431	0.1543	26.8511	0.8750
SARMAX	lightgray0.0703	lightgray0.0905	lightgray12.5076	0.8750	lightgray0.0794	lightgray0.0919	lightgray14.4916	0.8750
Holt-Winters	0.2231	0.2994	42.3489	0.8750	0.0996	0.1192	19.5066	0.8750
KARMA	0.2916	0.3200	56.9037	0.7500	0.1054	0.1170	19.6518	0.8750

The MKSARMAX model excels over the competing models in MAE and MAPE accuracy measures, except in the 8-steps forecasts, and follows 84.3100% of the time series directional movement in-sample and 100% in all out-of-sample steps. Specifically, the MAE and MAPE values of the fitted MKSARMAX model are approximately 37% and 46%, respectively, lower when compared to the ones from the KARMA model (in-sample). Although all models got the correct direction for the first-step forecast, MKSARMAX presented MAE, RMSE, and MAPE around 99% lower than the competing models. The MAE, RMSE, and MAPE values of the fitted MKSARMAX model when compared to the ones from βSARMA are about 44%, 22%, and 42%, respectively, lower for the cumulative 8-steps traditional forecast, and about 38%, 12%, and 36% lower for the rolling window forecast.

**Fig 5:**
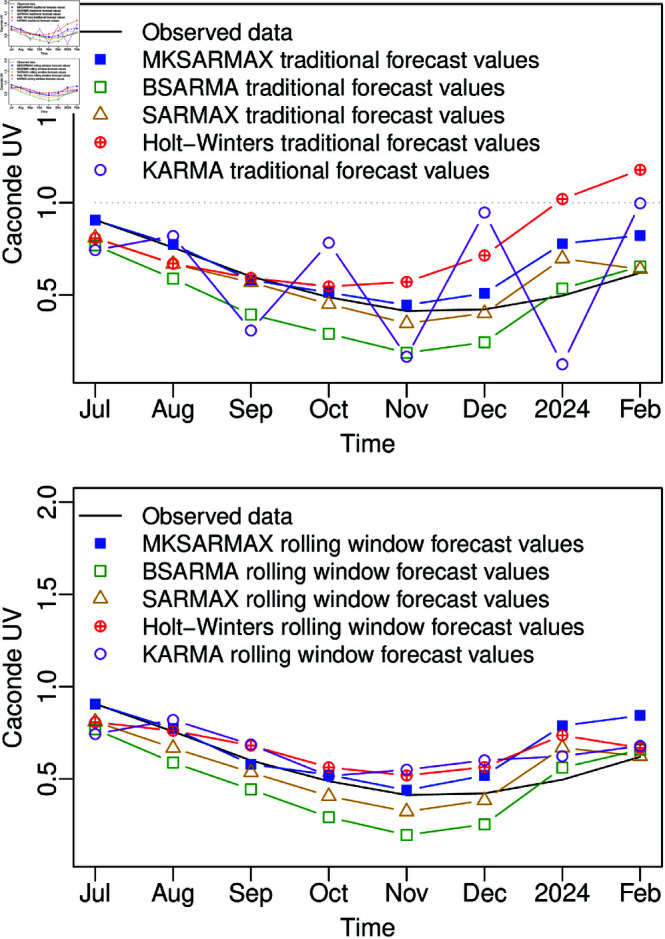
Eight steps out-of-sample traditional and rolling window forecasts comparison of MKSARMAX, βSARMA, SARMAX, additive Holt-Winters, and KARMA models for the Caconde UV dataset.

For a qualitative analysis, Fig 5 displays the observed and forecasted values for 8 steps out-of-sample. It is noted that the traditional and rolling window forecasts of MKSARMAX, βSARMA, and SARMAX are very close for this dataset. Besides the Holt-Winters and KARMA models having improved in the rolling window forecast compared to the traditional forecast, the MKSARMAX still presented the best values in most accuracy measures (Table 5). It can be highlighted that besides SARMAX and additive Holt-Winters presenting the second and third-best measures, respectively, in most of the forecast accuracy measures, these models predicted values above 1, exceeding the variable range, emphasizing the importance of a judicious model selection for reliable seasonal double-bounded time series modeling. Similar to the βSARMA and KARMA models, the MKSARMAX model accounts for the bounded nature of the response variable. Therefore, its superior performance relative to these models can be attributed to the greater flexibility of the MK distribution, or the inclusion of stochastic seasonality components – offering an advantage over the KARMA model, or the incorporation of exogenous regressors, which are not present in the βSARMA model, or a combination of these factors.

### 5.3 Application to Guarapiranga useful water volume dataset

**Fig 6:**
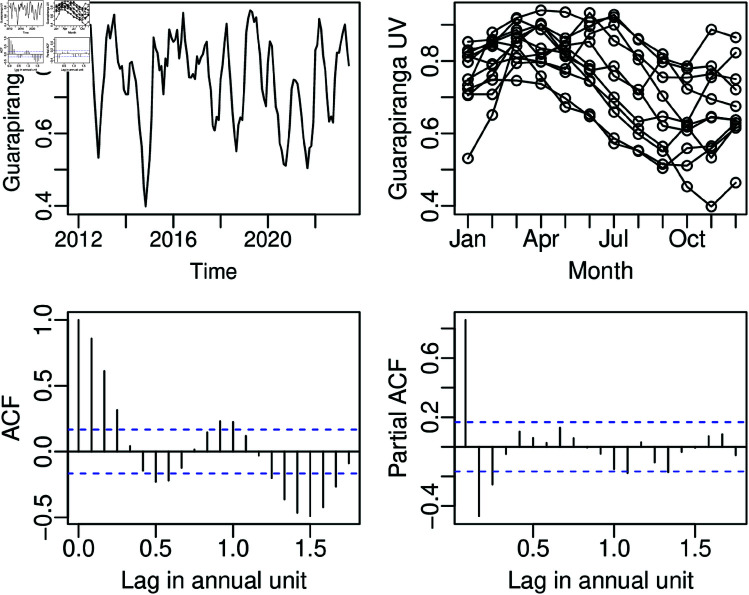
Time series, seasonal plot, ACF, and PACF for the Guarapiranga UV dataset from t=1 to 138.

The second dataset utilized to evaluate the effectiveness of the proposed model is the monthly useful water volume of the Guarapiranga Reservoir, situated on the border between Itapecerica da Serra and Embu-Guaçu, SP, Brazil. The Guarapiranga Reservoir is used as a source of public water supply. The dataset comprises Guarapiranga UV data collected monthly from January 2012 to June 2024, with the last 8 observations employed to assess the performance of the model forecasting for *n* = 138 and *H* = 8. The dataset is shown in Fig 6. The Guarapiranga UV data range from 0.3980 to 0.9400, with an unconditional median of 0.7760, an average of 0.7484, and a standard deviation of 0.1199. Fig 6 suggests the presence of seasonality with an annual frequency, a conclusion supported by the Friedman test (p-value<0.0001), which indicates evidence of a seasonal time series.

**Fig 7:**
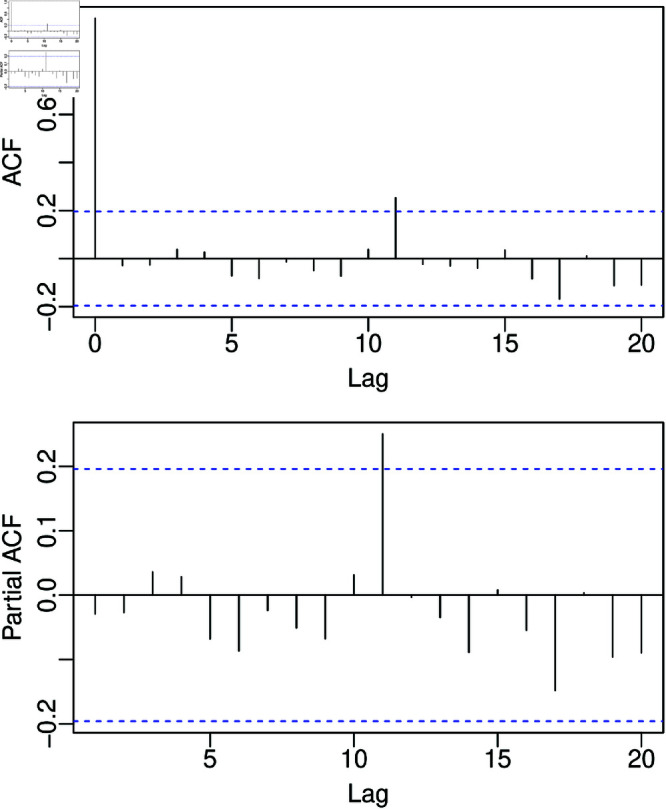
ACF and PACF of MKSARMAX $(1,2)\times (1,3{)}_{12}+X_{14}\beta_{1}+X_{21}\beta_{2}$ quantile residuals for the Guarapiranga UV dataset.

**Fig 8:**
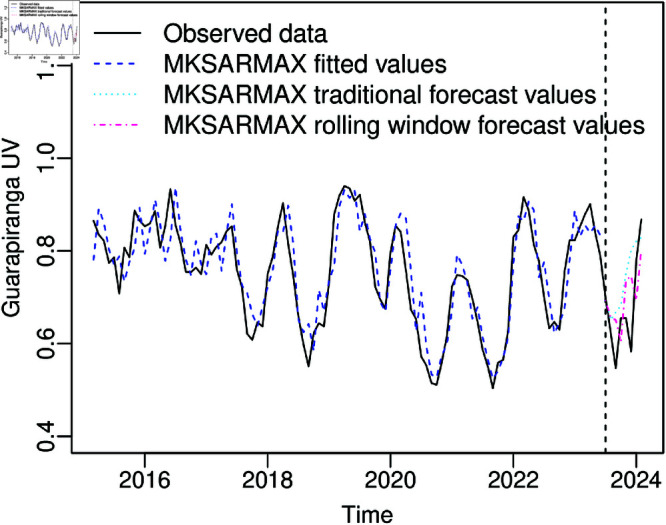
Fitted MKSARMAX $(1,2)\times (1,3{)}_{12}+X_{14}\beta_{1}+X_{21}\beta_{2}$ and 8 steps forecast for the Guarapiranga UV dataset.

The best model up to order 4, as determined by the methodology outlined in the previous application was found to be the MKSARMAX (1,2) × $(1,3{)}_{12}+X_{14}\beta_{1}+X_{21}\beta_{2}$, with the first and second lags of the seasonal moving-average term not considered and *X*_14_ and *X*_21_ being exogenous regressors changing the level of the time series in the Brazil water crises [48,51] from January 2014 to December 2014 and from January 2021 to December 2021, respectively, caused by drought periods. The drought periods led to low useful water volume in the reservoirs and compromised the population’s water supply. The fitted MKSARMAX (1,2) × $(1,3{)}_{12}+X_{14}\beta_{1}+X_{21}\beta_{2}$ model and the results of diagnostic tests are presented in Table 6. The tests outlined in Table 6 indicate an appropriate fit of the MKSARMAX model to the Guarapiranga UV data. The ACF and PACF of the residuals, presented in Fig 7, show most autocorrelation and partial autocorrelation within the 95% confidence interval, not rejecting the null hypothesis of non-autocorrelation. The significant $\phi_{1}$ informs that UV in the previous month helps significantly explain UV in the current month, and the significant $\Phi_{1}$ informs that UV in the same month of the previous year helps to explain UV in the current month. The significant $\theta_{1}$ and $\theta_{2}$ inform that the UV error predictions in the two previous months significantly help to explain UV in the current month, and significant $\Theta_{3}$ informs that the UV error prediction of the same month three years ago helps to explain the UV in the current month. Also, the lower useful water volume levels in the Guarapiranga Reservoir in the years 2014 and 2021 are significantly different from those in other periods, as the negative values of the $\beta_{1}$ and $\beta_{2}$ estimates are significantly different from zero. Fig 8 illustrates the observed, fitted, and forecast values of the MKSARMAX model. It is evident from Fig 8 that the MKSARMAX model adeptly captures the variability present in the observed dataset.

**Table 6:** Fitted MKSARMAX $(1,2)\times (1,3{)}_{12}+X_{14}\beta_{1}+X_{21}\beta_{2}$ for the Guarapiranga UV data. CMLE coefficients, confidence intervals, statistic and p-value of Wald test, and MBIC are presented as well as the statistics and p-value of Ljung-Box, Jarque-Bera, and ARCH tests.

Parameter	CMLE	Lower bound	Upper bound	Statistic	p-value
$\beta_{0}$	0.2838	0.1271	0.4406	3.5492	0.0004
$\beta_{1}$	–1.5112	–2.5266	–0.4958	–2.9170	0.0035
$\beta_{2}$	–0.3069	–0.4654	–0.1484	–3.7946	0.0001
$\phi_{1}$	0.6704	0.4815	0.8592	6.9569	<0.0001
$\theta_{1}$	–0.2316	–0.4491	–0.0140	–2.0866	0.0369
$\theta_{2}$	–0.3683	–0.5204	–0.2162	–4.7462	<0.0001
$\Phi_{1}$	0.4525	0.2477	0.6572	4.3314	<0.0001
$\Theta_{3}$	–0.3312	–0.5258	–0.1366	–3.3362	0.0008
α	12.0302	10.0868	13.9735	12.133	<0.0001
MBIC=−390.0307;					
Ljung-Box test statistic = 24.6649 (p-value =0.1719);					
Jarque-Bera test statistic = 4.7096 (p-value =0.0949);					
ARCH test statistic = 5.2504 (p-value =0.8738).					

**Fig 9:**
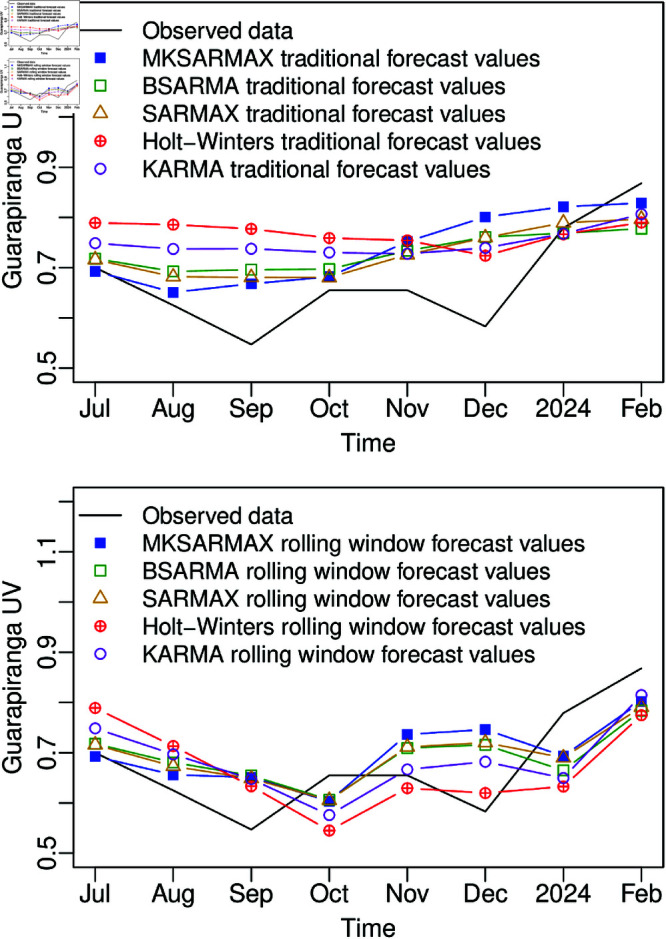
Eight steps out-of-sample traditional and rolling window forecasts comparison of MKSARMAX, βSARMA, SARMAX, additive Holt-Winters, and KARMA models for the Guarapiranga UV dataset.

The comparison between the MKSARMAX model and its competitors is presented in Tables 7 and 8. We fitted the SARMAX and the βSARMA models with the same autoregressive and moving-average terms as MKSARMAX, and for the KARMA model, the exogenous regressor $\cos\left(\frac{2\pi t}{12}\right)$ was added to model the seasonality. Considering the absolute value loss function, the Diebold-Mariano test indicates that the MKSARMAX model has a better traditional forecast than the Holt-Winters model (p-value = 0.0721). The MKSARMAX model demonstrates superior performance over the βSARMA, SARMAX, KARMA, and Holt-Winters models regarding most of the accuracy measures, except for the MAE, RMSE, and MAPE values in the in-sample fit, from 6 to 8-steps in the traditional forecast, and from 5 to 8-steps in the rolling window forecast. Specifically, for the in-sample prediction, the MDA value of the MKSARMAX model is about 37% higher, the MAE and RMSE values are approximately 32% lower, and the MAPE value is approximately 33% lower than those of the additive Holt-Winters method. For the first-step forecast, MKSARMAX presented MAE, RMSE, and MAPE values around 59% lower than the βSARMA model, 54% lower than the SARMAX model, 92% lower than the additive Holt-Winters method, and 85% lower than the KARMA model. For the cumulative 8-steps forecast, the MKSARMAX model presents the MAE, RMSE, and MAPE values approximately 37%, 24%, and 36%, respectively, lower than the additive Holt-Winters method in the traditional forecast and about 13%, 6%, and 7% in the rolling window forecast. Fig 9 illustrates the out-of-sample 8-steps forecast alongside the observed values, corroborating the evidence from Table 8 that the competing models get better rolling window forecasts than the traditional forecast, but not enough to surpass the MKSARMAX performance in the initial steps. We can attribute the better performance of the MKSARMAX in the initial steps over the competitors, especially the SARMAX model, to the flexibility of the MK distribution.

**Table 7:** In-sample prediction accuracy measures MAE, RMSE, MAPE, and MDA for MKSARMAX, βSARMA, SARMAX, additive Holt-Winters, and KARMA models fit of observations from t=39 to 138 of the Guarapiranga UV dataset. The best value for each measure is shaded light gray.

Model	MAE	RMSE	MAPE	MDA
MKSARMAX$(1,2)\times (1,3{)}_{12}+X_{14}\beta_{1}+X_{21}\beta_{2}$	0.0409	0.0504	5.4826	lightgray0.7100
βSARMA$(1,2)\times (1,3{)}_{12}$	0.0407	0.0518	5.5069	0.6600
SARMAX$(1,2)\times (1,3{)}_{12}+X_{14}\beta_{1}+X_{21}\beta_{2}$	lightgray0.0403	lightgray0.0494	lightgray5.4243	lightgray0.7100
Additive Holt-Winters	0.0603	0.0739	8.1523	0.5200
KARMA$(1,2)+X_{14}\beta_{1}+X_{21}\beta_{2}+\cos\frac{2\pi t}{12}\beta_{3}$	0.0437	0.0543	5.9229	0.5900

**Table 8:** Out-of-sample traditional and rolling window forecasts accuracy measures MAE, RMSE, MAPE, and MDA for MKSARMAX, βSARMA, SARMAX, additive Holt-Winters, and KARMA models forecast of the Guarapiranga UV dataset. The best value for each measure is shaded light gray.

Model	Traditional forecast				Rolling window forecast			
	MAE	RMSE	MAPE	MDA	MAE	RMSE	MAPE	MDA
1 step								
MKSARMAX	lightgray0.0073	lightgray0.0073	lightgray1.0420	lightgray1.0000	lightgray0.0073	lightgray0.0073	lightgray1.0420	lightgray1.0000
βSARMA	0.0179	0.0179	2.5539	lightgray1.0000	0.0179	0.0179	2.5539	lightgray1.0000
SARMAX	0.0159	0.0159	2.2704	lightgray1.0000	0.0159	0.0159	2.2704	lightgray1.0000
Holt-Winters	0.0891	0.0891	12.7304	0.0000	0.0891	0.0891	12.7304	0.0000
KARMA	0.0486	0.0486	6.9413	lightgray1.0000	0.0486	0.0486	6.9413	lightgray1.0000
2 steps								
MKSARMAX	lightgray0.0166	lightgray0.0190	lightgray2.5876	lightgray1.0000	lightgray0.0192	lightgray0.0226	lightgray3.0066	lightgray1.0000
βSARMA	0.0426	0.0493	6.6691	lightgray1.0000	0.0368	0.0414	5.7328	lightgray1.0000
SARMAX	0.0364	0.0418	5.6896	lightgray1.0000	0.0320	0.0358	4.9761	lightgray1.0000
Holt-Winters	0.1248	0.1298	19.2067	0.0000	0.0887	0.0887	13.4252	0.0000
KARMA	0.0804	0.0864	12.4442	0.5000	0.0602	0.0613	9.2200	lightgray1.0000
3 steps								
MKSARMAX	lightgray0.0513	lightgray0.0715	lightgray9.0935	lightgray0.6667	lightgray0.0475	lightgray0.0629	lightgray8.3467	lightgray0.6667
βSARMA	0.0781	0.0949	13.5189	lightgray0.6667	0.0604	0.0707	10.3766	lightgray0.6667
SARMAX	0.0688	0.0843	11.9347	lightgray0.6667	0.0552	0.0656	9.5121	lightgray0.6667
Holt-Winters	0.1599	0.1700	26.8301	0.0000	0.0879	0.0879	14.2106	0.0000
KARMA	0.1171	0.1307	19.9058	0.3333	0.0737	0.0767	12.2771	lightgray0.6667
4 steps								
MKSARMAX	lightgray0.0452	lightgray0.0634	lightgray7.8417	lightgray0.7500	lightgray0.0485	lightgray0.0602	lightgray8.2276	lightgray0.7500
βSARMA	0.0691	0.0848	11.7437	lightgray0.7500	0.0574	0.0659	9.6388	lightgray0.7500
SARMAX	0.0579	0.0741	9.9156	lightgray0.7500	0.0538	0.0620	9.0312	lightgray0.7500
Holt-Winters	0.1459	0.1561	24.0900	0.2500	0.0934	0.0939	14.8526	0.0000
KARMA	0.1066	0.1193	17.7972	0.5000	0.0750	0.0773	12.2230	lightgray0.7500
5 steps								
MKSARMAX	lightgray0.0555	lightgray0.0713	lightgray9.2311	lightgray0.6000	0.0551	0.0650	9.0692	lightgray0.6000
βSARMA	0.0710	0.0836	11.7956	lightgray0.6000	0.0568	0.0637	9.3723	lightgray0.6000
SARMAX	0.0605	0.0734	10.0886	lightgray0.6000	lightgray0.0543	lightgray0.0609	lightgray8.9481	lightgray0.6000
Holt-Winters	0.1366	0.1465	22.3018	0.2000	0.0798	0.0847	12.6605	0.0000
KARMA	0.0998	0.1115	16.4536	0.4000	0.0624	0.0693	10.1361	lightgray0.6000
6 steps								
MKSARMAX	0.0826	0.1102	13.9213	lightgray0.5000	0.0731	0.0892	12.2230	lightgray0.5000
βSARMA	0.0888	0.1053	14.9117	lightgray0.5000	0.0695	0.0795	11.6051	lightgray0.5000
SARMAX	lightgray0.0798	lightgray0.0985	lightgray13.4596	lightgray0.5000	lightgray0.0681	0.0789	11.3763	lightgray0.5000
Holt-Winters	0.1373	0.1456	22.6081	0.1667	0.0726	0.0788	11.5993	0.1667
KARMA	0.1092	0.1201	18.1768	0.3333	0.0685	lightgray0.0751	lightgray11.2800	lightgray0.5000
7 steps								
MKSARMAX	0.0768	0.1033	12.7060	lightgray0.5714	0.0749	0.0887	12.0471	lightgray0.5714
βSARMA	0.0776	0.0976	12.9719	lightgray0.5714	0.0759	0.0853	12.0415	lightgray0.5714
SARMAX	lightgray0.0699	lightgray0.0913	lightgray11.7251	lightgray0.5714	lightgray0.0711	lightgray0.0804	lightgray11.3816	lightgray0.5714
Holt-Winters	0.1195	0.1349	19.6082	0.2857	0.0832	0.0915	12.6258	0.2857
KARMA	0.0952	0.1113	15.7919	0.4286	0.0772	0.0850	12.0408	lightgray0.5714
8 steps								
MKSARMAX	0.0721	0.0976	11.6837	lightgray0.6250	0.0738	0.0862	11.4910	lightgray0.6250
βSARMA	0.0792	0.0967	12.6525	0.5000	0.0769	0.0852	11.7466	lightgray0.6250
SARMAX	lightgray0.0701	lightgray0.0890	lightgray11.2875	lightgray0.6250	lightgray0.0719	lightgray0.0800	lightgray11.0700	lightgray0.6250
Holt-Winters	0.1143	0.1291	18.2834	0.3750	0.0844	0.0918	12.3915	0.2500
KARMA	0.0910	0.1063	14.7036	0.5000	0.0742	0.0817	11.3026	lightgray0.6250

In summary, the MKSARMAX model was a reliable tool for predicting and forecasting both UV datasets, along with favorable outcomes in the Monte Carlo simulations. The numerical assessments indicate that the MKSARMAX model is competitive for modeling and forecasting double-bounded hydro-environmental time series, being able to accommodate stochastic seasonality and exogenous regressors, in addition of having predictions within the double-bounded support of the variable of interest. The improvements that MKSARMAX model brings to modeling and forecasting hydro-environmental time series are crucial in water resource management. The applications showed that the model more accurately forecasted the useful water volume in the reservoirs in the following months, which can lead to more effective strategies to prevent risk conditions to the water supply for the population.

## 6 Conclusion

To fulfill the gap in the literature on stochastic time series models, in which the traditional Gaussian-based ARMA model is the most popular, we proposed a model based on the MK distribution. Unlike the symmetric Gaussian distribution, which has a unbounded support over ℝ, the MK distribution accounts for asymmetry and features a double-bounded support. The MK distribution was chosen due to its superior performance compared to the beta and Kumaraswamy distributions in fitting the useful water volume of 37 reservoirs in [2]. Specifically, the MK and its reflected distribution fitted better for approximately 84% of the reservoirs, whereas the Kumaraswamy and beta distributions each performed best for only about 8% of the reservoirs. Given that the MK distribution is derived from a transformation of the Kumaraswamy distribution, and considering that the KARMA model, which has a structure to accommodate the presence of serial autocorrelation in the conditional median of Kumaraswamy-distributed time series, outperformed the βARMA model in fitting a relative humidity dataset [9], there is a strong indication that an ARMA model based on the MK distribution could be competitive with existing models. Furthermore, the proposed model incorporates both stochastic seasonality and exogenous regressors, distinguishing it from previously introduced models such as βSARMA and KARMA, and offering a broader set of features for time series modeling.

In this paper, we introduced the MKSARMAX model, designed for fitting and forecasting double-bounded hydro-environmental time series characterized by stochastic seasonal dynamics. Several hydro-environmental variables are not symmetric and present bounded support, being adequately modeled by models that consider these characteristics. Any improvement in forecasting these variables is valuable because its practical implications are related to better water resource management. An inference approach, out-of-sample forecasting, diagnostic check, and observed information matrix were tailored for the proposed model. We conducted extensive Monte Carlo simulations, comprising 5000 replications of synthetic hydro-environmental time series, to assess the performance of the conditional likelihood inferences, indicating the consistency of the conditional maximum likelihood estimators even in moderate sample sizes.

Additionally, we conducted two experiments with measured hydro-environmental time series to validate our model further. Monthly UV from two different Brazilian reservoirs than the ones analyzed in [2] were chosen to be fitted and forecasted by MKSARMAX and competitor models. Overall, the proposed model outperformed competing models such as SARMAX, Holt-Winters, and even other models that consider double-bounded support data, such as KARMA and βSARMA models – similar to what was observed in [2], in terms of prediction and forecast accuracy. For instance, in the Caconde UV application for 1-step forecast, the derived MKSARMAX model demonstrated approximately 99% improvement in MAE, RMSE, and MAPE values, compared to the βSARMA and KARMA models, while in the Guarapiranga UV application, it exhibited a 92% better MAE, RMSE, and MAPE values compared to the additive Holt-Winters method. The accurate UV forecast in the following months can assist in the monitoring of the risk of floods and droughts, and management can take actions to attenuate the impact on water supply and power generation. Those findings corroborate the evidence in [2] for MK to be considered a good distribution to fit hydro-environmental variables.

Despite its advantages, the MKSARMAX model presents certain limitations. First, the process of identifying an optimal specification among the various possible model configurations is computationally intensive. Second, the model assumes that issues such as missing data and outliers are addressed prior to estimation, requiring user intervention. In light of these limitations, future research could focus on developing a robust version of the model capable of handling outliers more effectively. Additionally, the integration of machine learning-based hybrid approaches may offer further improvements in model performance. Another promising direction is the incorporation of prediction intervals, which would complement the point forecasts currently provided by the model and offer a more comprehensive assessment of forecast uncertainty.

In conclusion, our study highlights the effectiveness of the proposed MKSARMAX model as a valuable tool for fitting and forecasting double-bounded hydro-environmental data. The model applied to the useful water volume datasets indicates that the MKSARMAX can offer substantial contributions to the field of hydro-environmental stochastic modeling.

## Supporting information

S1 Appendix Observed information matrix (PDF)

S2 Appendix Supplementary simulation results. (PDF)
