# Supplementary material for: Modified Kumaraswamy seasonal autoregressive moving average models with exogenous regressors for double-bounded hydro-environmental data
Source: PLoS One. 2025 May 20;20(5):e0324721. doi: 10.1371/journal.pone.0324721 (PMC12091792; doi:10.1371/journal.pone.0324721)
Supplement: S1 Appendix — (PDF) [file pone.0324721.s001.pdf]

S1 Appendix.

Observed information matrix

In this appendix, we provide the second derivatives of the conditional log-likelihood function with respect to the parameters and observed information matrix, which will be used to obtain the asymptotic variance of the estimators in the hypothesis testing inference and confidence intervals for the MKSARMAX model parameters. The derivatives of $\frac{\partial \ell_t(\mu_t,\alpha)}{\partial \mu_t}$ with respect to μ_t and α are given by

$$\frac{\partial^2 \ell_t(\mu_t,\alpha)}{\partial \mu_t^2} = \frac{\alpha e^{\alpha}\left[2\mu_t e^{\alpha} + (\alpha - 2\mu_t)e^{\alpha/\mu_t}\right]}{\mu_t^4(\mu_t^{\triangle})^2\mu_t^{\star}} + \frac{2\alpha^2 e^{2\alpha}\log(0.5)y_t^{\star}}{\mu_t^4(\mu_t^{\triangle})^2(\mu_t^{\star})^3} + \frac{\alpha e^{\alpha}\left\{\left[2\mu_t e^{\alpha} + (\alpha - 2\mu_t)e^{\alpha/\mu_t}\right]\log(0.5)y_t^{\star} + \alpha e^{\alpha}\right\}}{\mu_t^4(\mu_t^{\triangle})^2(\mu_t^{\star})^2},$$

$$\frac{\partial^2 \ell_t(\mu_t,\alpha)}{\partial \alpha \partial \mu_t} = \frac{\partial}{\partial \alpha}\left(\frac{\partial \ell_t(\mu_t,\alpha)}{\partial \mu_t}\right) = \frac{e^{\alpha}\log(0.5)\left[\mu_t(-e^{\alpha})\alpha(y_t-1)\mu_t^{\triangle}\right]}{\mu_t^3 y_t(\mu_t^{\triangle})^2 y_t^{\triangle}(\mu_t^{\star})^2} - \frac{e^{\alpha}\log(0.5)\left[\mu_t e^{\alpha} - (\mu_t\alpha + \mu_t - \alpha)e^{\alpha/\mu_t}\right]y_t^{\star}}{\mu_t^3(\mu_t^{\triangle})^2(\mu_t^{\star})^2} + \frac{(\mu_t-1)\alpha e^{2\alpha}}{\mu_t^3(\mu_t^{\triangle})^2(\mu_t^{\star})^2} + \frac{2\alpha e^{2\alpha}(\mu_t-1)\log(0.5)y_t^{\star}}{\mu_t^3(\mu_t^{\triangle})^2(\mu_t^{\star})^3} - \frac{e^{\alpha}\left[\mu_t e^{\alpha} - (\mu_t\alpha + \mu_t - \alpha)e^{\alpha/\mu_t}\right]}{\mu_t^3(\mu_t^{\triangle})^2\mu_t^{\star}},$$

where $\mu_t^{\triangle} = e^{\alpha} - e^{\alpha/\mu_t}$ and $y_t^{\triangle} = e^{\alpha} - e^{\alpha/y_t}$.

For $\gamma_h = \alpha$, the second derivative of $\ell_t(\mu_t,\alpha)$ with respect to the parameter α is given by

$$\frac{\partial^2 \ell_t(\mu_t,\alpha)}{\partial \alpha^2} = \frac{2(\mu_t-1)e^{2\alpha}(y_t-1)\log(0.5)}{\mu_t y_t \mu_t^{\triangle}\left(e^{\alpha/y_t} - e^{\alpha}\right)(\mu_t^{\star})^2} + \frac{2(\mu_t-1)^2 e^{2\alpha}\log(0.5)y_t^{\star}}{\mu_t^2\left(\mu_t^{\triangle}\right)^2(\mu_t^{\star})^3} + \frac{\varphi_t(\mu_t-1)\log(0.5)y_t^{\star}}{\mu_t\mu_t^{\star}} + \frac{(\mu_t-1)^2 e^{2\alpha}\log(0.5)y_t^{\star}}{\mu_t^2\left(\mu_t^{\triangle}\right)^2(\mu_t^{\star})^2} + \frac{(\mu_t-1)^2 e^{2\alpha}}{\mu_t^2\left(\mu_t^{\triangle}\right)^2(\mu_t^{\star})^2} + \frac{\varphi_t(\mu_t-1)}{\mu_t} + \frac{(\mu_t-1)^2 e^{2\alpha}}{\mu_t^2\left(\mu_t^{\triangle}\right)^2\mu_t^{\star}} - \frac{(y_t-1)^2 e^{\alpha-\alpha/y_t}\left(\log(0.5)/\mu_t^{\star} - 1\right)}{y_t^2\left(1 - e^{\alpha-\alpha/y_t}\right)} - \frac{(y_t-1)^2 e^{2\alpha-2\alpha/y_t}\left(\log(0.5)/\mu_t^{\star} - 1\right)}{y_t^2\left(e^{\alpha-\alpha/y_t} - 1\right)^2} - \frac{1}{\alpha^2}.$$

The observed information matrix $\mathbf{K}$ is given by

$$\mathbf{K}(\boldsymbol{\gamma}) = \begin{bmatrix} \mathrm{K}_{(\beta_0,\beta_0)} & \mathrm{K}_{(\beta_0,\boldsymbol{\beta})} & \mathrm{K}_{(\beta_0,\boldsymbol{\phi})} & \mathrm{K}_{(\beta_0,\boldsymbol{\theta})} & \mathrm{K}_{(\beta_0,\boldsymbol{\Phi})} & \mathrm{K}_{(\beta_0,\boldsymbol{\Theta})} & \mathrm{K}_{(\beta_0,\alpha)} \\ \mathrm{K}_{(\boldsymbol{\beta},\beta_0)} & \mathrm{K}_{(\boldsymbol{\beta},\boldsymbol{\beta})} & \mathrm{K}_{(\boldsymbol{\beta},\boldsymbol{\phi})} & \mathrm{K}_{(\boldsymbol{\beta},\boldsymbol{\theta})} & \mathrm{K}_{(\boldsymbol{\beta},\boldsymbol{\Phi})} & \mathrm{K}_{(\boldsymbol{\beta},\boldsymbol{\Theta})} & \mathrm{K}_{(\boldsymbol{\beta},\alpha)} \\ \mathrm{K}_{(\boldsymbol{\phi},\beta_0)} & \mathrm{K}_{(\boldsymbol{\phi},\boldsymbol{\beta})} & \mathrm{K}_{(\boldsymbol{\phi},\boldsymbol{\phi})} & \mathrm{K}_{(\boldsymbol{\phi},\boldsymbol{\theta})} & \mathrm{K}_{(\boldsymbol{\phi},\boldsymbol{\Phi})} & \mathrm{K}_{(\boldsymbol{\phi},\boldsymbol{\Theta})} & \mathrm{K}_{(\boldsymbol{\phi},\alpha)} \\ \mathrm{K}_{(\boldsymbol{\theta},\beta_0)} & \mathrm{K}_{(\boldsymbol{\theta},\boldsymbol{\beta})} & \mathrm{K}_{(\boldsymbol{\theta},\boldsymbol{\phi})} & \mathrm{K}_{(\boldsymbol{\theta},\boldsymbol{\theta})} & \mathrm{K}_{(\boldsymbol{\theta},\boldsymbol{\Phi})} & \mathrm{K}_{(\boldsymbol{\theta},\boldsymbol{\Theta})} & \mathrm{K}_{(\boldsymbol{\theta},\alpha)} \\ \mathrm{K}_{(\boldsymbol{\Phi},\beta_0)} & \mathrm{K}_{(\boldsymbol{\Phi},\boldsymbol{\beta})} & \mathrm{K}_{(\boldsymbol{\Phi},\boldsymbol{\phi})} & \mathrm{K}_{(\boldsymbol{\Phi},\boldsymbol{\theta})} & \mathrm{K}_{(\boldsymbol{\Phi},\boldsymbol{\Phi})} & \mathrm{K}_{(\boldsymbol{\Phi},\boldsymbol{\Theta})} & \mathrm{K}_{(\boldsymbol{\Phi},\alpha)} \\ \mathrm{K}_{(\boldsymbol{\Theta},\beta_0)} & \mathrm{K}_{(\boldsymbol{\Theta},\boldsymbol{\beta})} & \mathrm{K}_{(\boldsymbol{\Theta},\boldsymbol{\phi})} & \mathrm{K}_{(\boldsymbol{\Theta},\boldsymbol{\theta})} & \mathrm{K}_{(\boldsymbol{\Theta},\boldsymbol{\Phi})} & \mathrm{K}_{(\boldsymbol{\Theta},\boldsymbol{\Theta})} & \mathrm{K}_{(\boldsymbol{\Theta},\alpha)} \\ \mathrm{K}_{(\alpha,\beta_0)} & \mathrm{K}_{(\alpha,\boldsymbol{\beta})} & \mathrm{K}_{(\alpha,\boldsymbol{\phi})} & \mathrm{K}_{(\alpha,\boldsymbol{\theta})} & \mathrm{K}_{(\alpha,\boldsymbol{\Phi})} & \mathrm{K}_{(\alpha,\boldsymbol{\Theta})} & \mathrm{K}_{(\alpha,\alpha)} \end{bmatrix},$$

where

$$\begin{aligned}
\mathrm{K}(\beta_0, \beta_0) = &-\Bigg\{ \left(\frac{\partial g(\mu)}{\partial \beta_0}\right)^\top \frac{\partial^2 \ell(\mu,\alpha)}{\partial \mu^2} \left(\frac{\partial \mu}{\partial g(\mu)}\right)^2 \frac{\partial g(\mu)}{\partial \beta_0} + \\
&\left(\frac{\partial g(\mu)}{\partial \beta_0}\right)^\top \frac{\partial \ell(\mu,\alpha)}{\partial \mu} \left[\frac{\partial}{\partial \mu}\left(\frac{\partial \mu}{\partial g(\mu)}\right)\right] \frac{\partial g(\mu)}{\partial \beta_0} + \\
&(\mathbb{1})^\top \frac{\partial \ell(\mu,\alpha)}{\partial \mu} \frac{\partial \mu}{\partial g(\mu)} \frac{\partial^2 g(\mu)}{\partial \beta_0^2} \Bigg\}, \\
\mathrm{K}(\beta_0, \boldsymbol{\beta}) = \mathrm{K}(\boldsymbol{\beta}, \beta_0)^\top = &-\Bigg\{ \left(\frac{\partial g(\mu)}{\partial \beta_0}\right)^\top \frac{\partial^2 \ell(\mu,\alpha)}{\partial \mu^2} \left(\frac{\partial \mu}{\partial g(\mu)}\right)^2 \frac{\partial g(\mu)}{\partial \boldsymbol{\beta}} + \\
&\left(\frac{\partial g(\mu)}{\partial \beta_0}\right)^\top \frac{\partial \ell(\mu,\alpha)}{\partial \mu} \left[\frac{\partial}{\partial \mu}\left(\frac{\partial \mu}{\partial g(\mu)}\right)\right] \frac{\partial g(\mu)}{\partial \boldsymbol{\beta}} + \\
&(\mathbb{1})^\top \frac{\partial \ell(\mu,\alpha)}{\partial \mu} \frac{\partial \mu}{\partial g(\mu)} \frac{\partial^2 g(\mu)}{\partial \beta_0 \partial \boldsymbol{\beta}} \Bigg\}, \\
\mathrm{K}(\beta_0, \boldsymbol{\phi}) = \mathrm{K}(\boldsymbol{\phi}, \beta_0)^\top = &-\Bigg\{ \left(\frac{\partial g(\mu)}{\partial \beta_0}\right)^\top \frac{\partial^2 \ell(\mu,\alpha)}{\partial \mu^2} \left(\frac{\partial \mu}{\partial g(\mu)}\right)^2 \frac{\partial g(\mu)}{\partial \boldsymbol{\phi}} + \\
&\left(\frac{\partial g(\mu)}{\partial \beta_0}\right)^\top \frac{\partial \ell(\mu,\alpha)}{\partial \mu} \left[\frac{\partial}{\partial \mu}\left(\frac{\partial \mu}{\partial g(\mu)}\right)\right] \frac{\partial g(\mu)}{\partial \boldsymbol{\phi}} + \\
&(\mathbb{1})^\top \frac{\partial \ell(\mu,\alpha)}{\partial \mu} \frac{\partial \mu}{\partial g(\mu)} \frac{\partial^2 g(\mu)}{\partial \beta_0 \partial \boldsymbol{\phi}} \Bigg\}, \\
\mathrm{K}(\beta_0, \boldsymbol{\theta}) = \mathrm{K}(\boldsymbol{\theta}, \beta_0)^\top = &-\Bigg\{ \left(\frac{\partial g(\mu)}{\partial \beta_0}\right)^\top \frac{\partial^2 \ell(\mu,\alpha)}{\partial \mu^2} \left(\frac{\partial \mu}{\partial g(\mu)}\right)^2 \frac{\partial g(\mu)}{\partial \boldsymbol{\theta}} + \\
&\left(\frac{\partial g(\mu)}{\partial \beta_0}\right)^\top \frac{\partial \ell(\mu,\alpha)}{\partial \mu} \left[\frac{\partial}{\partial \mu}\left(\frac{\partial \mu}{\partial g(\mu)}\right)\right] \frac{\partial g(\mu)}{\partial \boldsymbol{\theta}} + \\
&(\mathbb{1})^\top \frac{\partial \ell(\mu,\alpha)}{\partial \mu} \frac{\partial \mu}{\partial g(\mu)} \frac{\partial^2 g(\mu)}{\partial \beta_0 \partial \boldsymbol{\theta}} \Bigg\}, \\
\mathrm{K}(\beta_0, \boldsymbol{\Phi}) = \mathrm{K}(\boldsymbol{\Phi}, \beta_0)^\top = &-\Bigg\{ \left(\frac{\partial g(\mu)}{\partial \beta_0}\right)^\top \frac{\partial^2 \ell(\mu,\alpha)}{\partial \mu^2} \left(\frac{\partial \mu}{\partial g(\mu)}\right)^2 \frac{\partial g(\mu)}{\partial \boldsymbol{\Phi}} + \\
&\left(\frac{\partial g(\mu)}{\partial \beta_0}\right)^\top \frac{\partial \ell(\mu,\alpha)}{\partial \mu} \left[\frac{\partial}{\partial \mu}\left(\frac{\partial \mu}{\partial g(\mu)}\right)\right] \frac{\partial g(\mu)}{\partial \boldsymbol{\Phi}} + \\
&(\mathbb{1})^\top \frac{\partial \ell(\mu,\alpha)}{\partial \mu} \frac{\partial \mu}{\partial g(\mu)} \frac{\partial^2 g(\mu)}{\partial \beta_0 \partial \boldsymbol{\Phi}} \Bigg\}, \\
\mathrm{K}(\beta_0, \boldsymbol{\Theta}) = \mathrm{K}(\boldsymbol{\Theta}, \beta_0)^\top = &-\Bigg\{ \left(\frac{\partial g(\mu)}{\partial \beta_0}\right)^\top \frac{\partial^2 \ell(\mu,\alpha)}{\partial \mu^2} \left(\frac{\partial \mu}{\partial g(\mu)}\right)^2 \frac{\partial g(\mu)}{\partial \boldsymbol{\Theta}} + \\
&\left(\frac{\partial g(\mu)}{\partial \beta_0}\right)^\top \frac{\partial \ell(\mu,\alpha)}{\partial \mu} \left[\frac{\partial}{\partial \mu}\left(\frac{\partial \mu}{\partial g(\mu)}\right)\right] \frac{\partial g(\mu)}{\partial \boldsymbol{\Theta}} + \\
&(\mathbb{1})^\top \frac{\partial \ell(\mu,\alpha)}{\partial \mu} \frac{\partial \mu}{\partial g(\mu)} \frac{\partial^2 g(\mu)}{\partial \beta_0 \partial \boldsymbol{\Theta}} \Bigg\}, \\
\mathrm{K}(\beta_0, \alpha) = \mathrm{K}(\alpha, \beta_0)^\top = &-\left(\frac{\partial g(\mu)}{\partial \beta_0}\right)^\top \frac{\partial^2 \ell(\mu,\alpha)}{\partial \alpha \partial \mu} \frac{\partial \mu}{\partial g(\mu)} \mathbb{1}, \\
\mathrm{K}(\boldsymbol{\beta}, \boldsymbol{\beta}) = &-\Bigg\{ \left(\frac{\partial g(\mu)}{\partial \boldsymbol{\beta}}\right)^\top \frac{\partial^2 \ell(\mu,\alpha)}{\partial \mu^2} \left(\frac{\partial \mu}{\partial g(\mu)}\right)^2 \frac{\partial g(\mu)}{\partial \boldsymbol{\beta}} +
\end{aligned}$$

$$\left(\frac{\partial g(\mu)}{\partial \boldsymbol{\beta}}\right)^{\top} \frac{\partial \ell(\mu,\alpha)}{\partial \mu} \left[\frac{\partial}{\partial \mu}\left(\frac{\partial \mu}{\partial g(\mu)}\right)\right] \frac{\partial g(\mu)}{\partial \boldsymbol{\beta}} +$$

$$(\mathbb{1})^{\top} \frac{\partial \ell(\mu,\alpha)}{\partial \mu} \frac{\partial \mu}{\partial g(\mu)} \frac{\partial^2 g(\mu)}{\partial \boldsymbol{\beta}^2} \Bigg\},$$

$$\mathrm{K}(\boldsymbol{\beta},\boldsymbol{\phi}) = \mathrm{K}(\boldsymbol{\phi},\boldsymbol{\beta})^{\top} = -\Bigg\{ \left(\frac{\partial g(\mu)}{\partial \boldsymbol{\beta}}\right)^{\top} \frac{\partial^2 \ell(\mu,\alpha)}{\partial \mu^2} \left(\frac{\partial \mu}{\partial g(\mu)}\right)^2 \frac{\partial g(\mu)}{\partial \boldsymbol{\phi}} +$$

$$\left(\frac{\partial g(\mu)}{\partial \boldsymbol{\beta}}\right)^{\top} \frac{\partial \ell(\mu,\alpha)}{\partial \mu} \left[\frac{\partial}{\partial \mu}\left(\frac{\partial \mu}{\partial g(\mu)}\right)\right] \frac{\partial g(\mu)}{\partial \boldsymbol{\phi}} +$$

$$(\mathbb{1})^{\top} \frac{\partial \ell(\mu,\alpha)}{\partial \mu} \frac{\partial \mu}{\partial g(\mu)} \frac{\partial^2 g(\mu)}{\partial \boldsymbol{\beta} \partial \boldsymbol{\phi}} \Bigg\},$$

$$\mathrm{K}(\boldsymbol{\beta},\boldsymbol{\theta}) = \mathrm{K}(\boldsymbol{\theta},\boldsymbol{\beta})^{\top} = -\Bigg\{ \left(\frac{\partial g(\mu)}{\partial \boldsymbol{\beta}}\right)^{\top} \frac{\partial^2 \ell(\mu,\alpha)}{\partial \mu^2} \left(\frac{\partial \mu}{\partial g(\mu)}\right)^2 \frac{\partial g(\mu)}{\partial \boldsymbol{\theta}} +$$

$$\left(\frac{\partial g(\mu)}{\partial \boldsymbol{\beta}}\right)^{\top} \frac{\partial \ell(\mu,\alpha)}{\partial \mu} \left[\frac{\partial}{\partial \mu}\left(\frac{\partial \mu}{\partial g(\mu)}\right)\right] \frac{\partial g(\mu)}{\partial \boldsymbol{\theta}} +$$

$$(\mathbb{1})^{\top} \frac{\partial \ell(\mu,\alpha)}{\partial \mu} \frac{\partial \mu}{\partial g(\mu)} \frac{\partial^2 g(\mu)}{\partial \boldsymbol{\beta} \partial \boldsymbol{\theta}} \Bigg\},$$

$$\mathrm{K}(\boldsymbol{\beta},\boldsymbol{\Phi}) = \mathrm{K}(\boldsymbol{\Phi},\boldsymbol{\beta})^{\top} = -\Bigg\{ \left(\frac{\partial g(\mu)}{\partial \boldsymbol{\beta}}\right)^{\top} \frac{\partial^2 \ell(\mu,\alpha)}{\partial \mu^2} \left(\frac{\partial \mu}{\partial g(\mu)}\right)^2 \frac{\partial g(\mu)}{\partial \boldsymbol{\Phi}} +$$

$$\left(\frac{\partial g(\mu)}{\partial \boldsymbol{\beta}}\right)^{\top} \frac{\partial \ell(\mu,\alpha)}{\partial \mu} \left[\frac{\partial}{\partial \mu}\left(\frac{\partial \mu}{\partial g(\mu)}\right)\right] \frac{\partial g(\mu)}{\partial \boldsymbol{\Phi}} +$$

$$(\mathbb{1})^{\top} \frac{\partial \ell(\mu,\alpha)}{\partial \mu} \frac{\partial \mu}{\partial g(\mu)} \frac{\partial^2 g(\mu)}{\partial \boldsymbol{\beta} \partial \boldsymbol{\Phi}} \Bigg\},$$

$$\mathrm{K}(\boldsymbol{\beta},\boldsymbol{\Theta}) = \mathrm{K}(\boldsymbol{\Theta},\boldsymbol{\beta})^{\top} = -\Bigg\{ \left(\frac{\partial g(\mu)}{\partial \boldsymbol{\beta}}\right)^{\top} \frac{\partial^2 \ell(\mu,\alpha)}{\partial \mu^2} \left(\frac{\partial \mu}{\partial g(\mu)}\right)^2 \frac{\partial g(\mu)}{\partial \boldsymbol{\Theta}} +$$

$$\left(\frac{\partial g(\mu)}{\partial \boldsymbol{\beta}}\right)^{\top} \frac{\partial \ell(\mu,\alpha)}{\partial \mu} \left[\frac{\partial}{\partial \mu}\left(\frac{\partial \mu}{\partial g(\mu)}\right)\right] \frac{\partial g(\mu)}{\partial \boldsymbol{\Theta}} +$$

$$(\mathbb{1})^{\top} \frac{\partial \ell(\mu,\alpha)}{\partial \mu} \frac{\partial \mu}{\partial g(\mu)} \frac{\partial^2 g(\mu)}{\partial \boldsymbol{\beta} \partial \boldsymbol{\Theta}} \Bigg\},$$

$$\mathrm{K}(\boldsymbol{\beta},\alpha) = \mathrm{K}(\alpha,\boldsymbol{\beta})^{\top} = -\left(\frac{\partial g(\mu)}{\partial \boldsymbol{\beta}}\right)^{\top} \frac{\partial^2 \ell(\mu,\alpha)}{\partial \alpha \partial \mu} \frac{\partial \mu}{\partial g(\mu)} \mathbb{1},$$

$$\mathrm{K}(\boldsymbol{\phi},\boldsymbol{\phi}) = -\Bigg\{ \left(\frac{\partial g(\mu)}{\partial \boldsymbol{\phi}}\right)^{\top} \frac{\partial^2 \ell(\mu,\alpha)}{\partial \mu^2} \left(\frac{\partial \mu}{\partial g(\mu)}\right)^2 \frac{\partial g(\mu)}{\partial \boldsymbol{\phi}} +$$

$$\left(\frac{\partial g(\mu)}{\partial \boldsymbol{\phi}}\right)^{\top} \frac{\partial \ell(\mu,\alpha)}{\partial \mu} \left[\frac{\partial}{\partial \mu}\left(\frac{\partial \mu}{\partial g(\mu)}\right)\right] \frac{\partial g(\mu)}{\partial \boldsymbol{\phi}} +$$

$$(\mathbb{1})^{\top} \frac{\partial \ell(\mu,\alpha)}{\partial \mu} \frac{\partial \mu}{\partial g(\mu)} \frac{\partial^2 g(\mu)}{\partial \boldsymbol{\phi}^2} \Bigg\},$$

$$\mathrm{K}(\boldsymbol{\phi},\boldsymbol{\theta}) = \mathrm{K}(\boldsymbol{\theta},\boldsymbol{\phi})^{\top} = -\Bigg\{ \left(\frac{\partial g(\mu)}{\partial \boldsymbol{\phi}}\right)^{\top} \frac{\partial^2 \ell(\mu,\alpha)}{\partial \mu^2} \left(\frac{\partial \mu}{\partial g(\mu)}\right)^2 \frac{\partial g(\mu)}{\partial \boldsymbol{\theta}} +$$

$$\left(\frac{\partial g(\mu)}{\partial \boldsymbol{\phi}}\right)^{\top} \frac{\partial \ell(\mu,\alpha)}{\partial \mu} \left[\frac{\partial}{\partial \mu}\left(\frac{\partial \mu}{\partial g(\mu)}\right)\right] \frac{\partial g(\mu)}{\partial \boldsymbol{\theta}} +$$

$$
\begin{aligned}
&(\mathbb{1})^\top \frac{\partial \ell(\mu,\alpha)}{\partial \mu}\frac{\partial \mu}{\partial g(\mu)}\frac{\partial^2 g(\mu)}{\partial \boldsymbol{\phi}\partial \boldsymbol{\theta}}\Bigg\},\\
\mathrm{K}(\boldsymbol{\phi},\boldsymbol{\Phi}) = \mathrm{K}(\boldsymbol{\Phi},\boldsymbol{\phi})^\top = &-\Bigg\{\left(\frac{\partial g(\mu)}{\partial \boldsymbol{\phi}}\right)^\top \frac{\partial^2 \ell(\mu,\alpha)}{\partial \mu^2}\left(\frac{\partial \mu}{\partial g(\mu)}\right)^2 \frac{\partial g(\mu)}{\partial \boldsymbol{\Phi}}+\\
&\left(\frac{\partial g(\mu)}{\partial \boldsymbol{\phi}}\right)^\top \frac{\partial \ell(\mu,\alpha)}{\partial \mu}\left[\frac{\partial}{\partial \mu}\left(\frac{\partial \mu}{\partial g(\mu)}\right)\right]\frac{\partial g(\mu)}{\partial \boldsymbol{\Phi}}+\\
&(\mathbb{1})^\top \frac{\partial \ell(\mu,\alpha)}{\partial \mu}\frac{\partial \mu}{\partial g(\mu)}\frac{\partial^2 g(\mu)}{\partial \boldsymbol{\phi}\partial \boldsymbol{\Phi}}\Bigg\},\\
\mathrm{K}(\boldsymbol{\phi},\boldsymbol{\Theta}) = \mathrm{K}(\boldsymbol{\Theta},\boldsymbol{\phi})^\top = &-\Bigg\{\left(\frac{\partial g(\mu)}{\partial \boldsymbol{\phi}}\right)^\top \frac{\partial^2 \ell(\mu,\alpha)}{\partial \mu^2}\left(\frac{\partial \mu}{\partial g(\mu)}\right)^2 \frac{\partial g(\mu)}{\partial \boldsymbol{\Theta}}+\\
&\left(\frac{\partial g(\mu)}{\partial \boldsymbol{\phi}}\right)^\top \frac{\partial \ell(\mu,\alpha)}{\partial \mu}\left[\frac{\partial}{\partial \mu}\left(\frac{\partial \mu}{\partial g(\mu)}\right)\right]\frac{\partial g(\mu)}{\partial \boldsymbol{\Theta}}+\\
&(\mathbb{1})^\top \frac{\partial \ell(\mu,\alpha)}{\partial \mu}\frac{\partial \mu}{\partial g(\mu)}\frac{\partial^2 g(\mu)}{\partial \boldsymbol{\phi}\partial \boldsymbol{\Theta}}\Bigg\},\\
\mathrm{K}(\boldsymbol{\phi},\alpha) = \mathrm{K}(\alpha,\boldsymbol{\phi})^\top = &-\left(\frac{\partial g(\mu)}{\partial \boldsymbol{\phi}}\right)^\top \frac{\partial^2 \ell(\mu,\alpha)}{\partial \alpha\partial \mu}\frac{\partial \mu}{\partial g(\mu)}\mathbb{1},\\
\mathrm{K}(\boldsymbol{\theta},\boldsymbol{\theta}) = &-\Bigg\{\left(\frac{\partial g(\mu)}{\partial \boldsymbol{\theta}}\right)^\top \frac{\partial^2 \ell(\mu,\alpha)}{\partial \mu^2}\left(\frac{\partial \mu}{\partial g(\mu)}\right)^2 \frac{\partial g(\mu)}{\partial \boldsymbol{\theta}}+\\
&\left(\frac{\partial g(\mu)}{\partial \boldsymbol{\theta}}\right)^\top \frac{\partial \ell(\mu,\alpha)}{\partial \mu}\left[\frac{\partial}{\partial \mu}\left(\frac{\partial \mu}{\partial g(\mu)}\right)\right]\frac{\partial g(\mu)}{\partial \boldsymbol{\theta}}+\\
&(\mathbb{1})^\top \frac{\partial \ell(\mu,\alpha)}{\partial \mu}\frac{\partial \mu}{\partial g(\mu)}\frac{\partial^2 g(\mu)}{\partial \boldsymbol{\theta}^2}\Bigg\},\\
\mathrm{K}(\boldsymbol{\theta},\boldsymbol{\Theta}) = \mathrm{K}(\boldsymbol{\Theta},\boldsymbol{\theta})^\top = &-\Bigg\{\left(\frac{\partial g(\mu)}{\partial \boldsymbol{\theta}}\right)^\top \frac{\partial^2 \ell(\mu,\alpha)}{\partial \mu^2}\left(\frac{\partial \mu}{\partial g(\mu)}\right)^2 \frac{\partial g(\mu)}{\partial \boldsymbol{\Theta}}+\\
&\left(\frac{\partial g(\mu)}{\partial \boldsymbol{\theta}}\right)^\top \frac{\partial \ell(\mu,\alpha)}{\partial \mu}\left[\frac{\partial}{\partial \mu}\left(\frac{\partial \mu}{\partial g(\mu)}\right)\right]\frac{\partial g(\mu)}{\partial \boldsymbol{\Theta}}+\\
&(\mathbb{1})^\top \frac{\partial \ell(\mu,\alpha)}{\partial \mu}\frac{\partial \mu}{\partial g(\mu)}\frac{\partial^2 g(\mu)}{\partial \boldsymbol{\theta}\partial \boldsymbol{\Theta}}\Bigg\},\\
\mathrm{K}(\boldsymbol{\theta},\alpha) = \mathrm{K}(\alpha,\boldsymbol{\theta})^\top = &-\left(\frac{\partial g(\mu)}{\partial \boldsymbol{\theta}}\right)^\top \frac{\partial^2 \ell(\mu,\alpha)}{\partial \alpha\partial \mu}\frac{\partial \mu}{\partial g(\mu)}\mathbb{1},\\
\mathrm{K}(\boldsymbol{\Phi},\boldsymbol{\theta}) = \mathrm{K}(\boldsymbol{\theta},\boldsymbol{\Phi})^\top = &-\Bigg\{\left(\frac{\partial g(\mu)}{\partial \boldsymbol{\Phi}}\right)^\top \frac{\partial^2 \ell(\mu,\alpha)}{\partial \mu^2}\left(\frac{\partial \mu}{\partial g(\mu)}\right)^2 \frac{\partial g(\mu)}{\partial \boldsymbol{\theta}}+\\
&\left(\frac{\partial g(\mu)}{\partial \boldsymbol{\Phi}}\right)^\top \frac{\partial \ell(\mu,\alpha)}{\partial \mu}\left[\frac{\partial}{\partial \mu}\left(\frac{\partial \mu}{\partial g(\mu)}\right)\right]\frac{\partial g(\mu)}{\partial \boldsymbol{\theta}}+\\
&(\mathbb{1})^\top \frac{\partial \ell(\mu,\alpha)}{\partial \mu}\frac{\partial \mu}{\partial g(\mu)}\frac{\partial^2 g(\mu)}{\partial \boldsymbol{\Phi}\partial \boldsymbol{\theta}}\Bigg\},\\
\mathrm{K}(\boldsymbol{\Phi},\boldsymbol{\Phi}) = &-\Bigg\{\left(\frac{\partial g(\mu)}{\partial \boldsymbol{\Phi}}\right)^\top \frac{\partial^2 \ell(\mu,\alpha)}{\partial \mu^2}\left(\frac{\partial \mu}{\partial g(\mu)}\right)^2 \frac{\partial g(\mu)}{\partial \boldsymbol{\Phi}}+\\
&\left(\frac{\partial g(\mu)}{\partial \boldsymbol{\Phi}}\right)^\top \frac{\partial \ell(\mu,\alpha)}{\partial \mu}\left[\frac{\partial}{\partial \mu}\left(\frac{\partial \mu}{\partial g(\mu)}\right)\right]\frac{\partial g(\mu)}{\partial \boldsymbol{\Phi}}+
\end{aligned}
$$

$$(\mathbb{1})^\top \frac{\partial \ell(\mu,\alpha)}{\partial \mu} \frac{\partial \mu}{\partial g(\mu)} \frac{\partial^2 g(\mu)}{\partial \boldsymbol{\Phi}^2} \Bigg\},$$

$$\mathrm{K}(\boldsymbol{\Phi},\boldsymbol{\Theta}) = \mathrm{K}(\boldsymbol{\Theta},\boldsymbol{\Phi})^\top = -\left\{ \left(\frac{\partial g(\mu)}{\partial \boldsymbol{\Phi}}\right)^\top \frac{\partial^2 \ell(\mu,\alpha)}{\partial \mu^2} \left(\frac{\partial \mu}{\partial g(\mu)}\right)^2 \frac{\partial g(\mu)}{\partial \boldsymbol{\Theta}} + \right.$$

$$\left(\frac{\partial g(\mu)}{\partial \boldsymbol{\Phi}}\right)^\top \frac{\partial \ell(\mu,\alpha)}{\partial \mu} \left[\frac{\partial}{\partial \mu}\left(\frac{\partial \mu}{\partial g(\mu)}\right)\right] \frac{\partial g(\mu)}{\partial \boldsymbol{\Theta}} +$$

$$\left.(\mathbb{1})^\top \frac{\partial \ell(\mu,\alpha)}{\partial \mu} \frac{\partial \mu}{\partial g(\mu)} \frac{\partial^2 g(\mu)}{\partial \boldsymbol{\Phi} \partial \boldsymbol{\Theta}} \right\},$$

$$\mathrm{K}(\boldsymbol{\Phi},\alpha) = \mathrm{K}(\alpha,\boldsymbol{\Phi})^\top = -\left(\frac{\partial g(\mu)}{\partial \boldsymbol{\Phi}}\right)^\top \frac{\partial^2 \ell(\mu,\alpha)}{\partial \alpha \partial \mu} \frac{\partial \mu}{\partial g(\mu)} \mathbb{1},$$

$$\mathrm{K}(\boldsymbol{\Theta},\boldsymbol{\Theta}) = -\left\{ \left(\frac{\partial g(\mu)}{\partial \boldsymbol{\Theta}}\right)^\top \frac{\partial^2 \ell(\mu,\alpha)}{\partial \mu^2} \left(\frac{\partial \mu}{\partial g(\mu)}\right)^2 \frac{\partial g(\mu)}{\partial \boldsymbol{\Theta}} + \right.$$

$$\left(\frac{\partial g(\mu)}{\partial \boldsymbol{\Theta}}\right)^\top \frac{\partial \ell(\mu,\alpha)}{\partial \mu} \left[\frac{\partial}{\partial \mu}\left(\frac{\partial \mu}{\partial g(\mu)}\right)\right] \frac{\partial g(\mu)}{\partial \boldsymbol{\Theta}} +$$

$$\left.(\mathbb{1})^\top \frac{\partial \ell(\mu,\alpha)}{\partial \mu} \frac{\partial \mu}{\partial g(\mu)} \frac{\partial^2 g(\mu)}{\partial \boldsymbol{\Theta}^2} \right\},$$

$$\mathrm{K}(\boldsymbol{\Theta},\alpha) = \mathrm{K}(\alpha,\boldsymbol{\Theta})^\top = -\left(\frac{\partial g(\mu)}{\partial \boldsymbol{\Theta}}\right)^\top \frac{\partial^2 \ell(\mu,\alpha)}{\partial \alpha \partial \mu} \frac{\partial \mu}{\partial g(\mu)} \mathbb{1},$$

$$\mathrm{K}(\alpha,\alpha) = -\frac{\partial^2 \ell(\mu,\alpha)}{\partial \alpha^2},$$

$\mathbb{1}$ is the $(n-\delta) \times 1$ vector of ones,

$$\frac{\partial}{\partial \mu}\left(\frac{\partial \mu}{\partial g(\mu)}\right) = -\,g''(\mu) \times \left(\frac{\partial \mu}{\partial g(\mu)}\right)^3 = -\frac{g''(\mu)}{(g'(\mu))^3},$$

$$\frac{\partial^2 g(\mu)}{\partial \beta_0^2} = \sum_{a=1}^{q} \theta_a \frac{\partial^2 g(\mu_{t-a})}{\partial \beta_0^2} + \sum_{b=1}^{Q} \Theta_b \frac{\partial^2 g(\mu_{t-bS})}{\partial \beta_0^2} -$$

$$\sum_{a=1}^{q} \sum_{b=1}^{Q} \theta_a \Theta_b \frac{\partial^2 g(\mu_{t-(a+bS)})}{\partial \beta_0^2},$$

$$\frac{\partial^2 g(\mu)}{\partial \beta_0 \partial \beta_c} = \sum_{a=1}^{q} \theta_a \frac{\partial^2 g(\mu_{t-a})}{\partial \beta_0 \partial \beta_c} + \sum_{b=1}^{Q} \Theta_b \frac{\partial^2 g(\mu_{t-bS})}{\partial \beta_0 \partial \beta_c} -$$

$$\sum_{a=1}^{q} \sum_{b=1}^{Q} \theta_a \Theta_b \frac{\partial^2 g(\mu_{t-(a+bS)})}{\partial \beta_0 \partial \beta_c},$$

$$\frac{\partial^2 g(\mu)}{\partial \beta_0 \partial \phi_i} = \sum_{a=1}^{q} \theta_a \frac{\partial^2 g(\mu_{t-a})}{\partial \beta_0 \partial \phi_i} + \sum_{b=1}^{Q} \Theta_b \frac{\partial^2 g(\mu_{t-bS})}{\partial \beta_0 \partial \phi_i} -$$

$$\sum_{a=1}^{q} \sum_{b=1}^{Q} \theta_a \Theta_b \frac{\partial^2 g(\mu_{t-(a+bS)})}{\partial \beta_0 \partial \phi_i},$$

$$\frac{\partial^2 g(\mu_t)}{\partial \beta_0 \partial \theta_a} = \frac{\partial}{\partial \beta_0}\left(\frac{\partial g(\mu_t)}{\partial \theta_a}\right) = \frac{\partial g(\mu_{t-a})}{\partial \beta_0} - \sum_{b=1}^{Q} \Theta_b \frac{\partial g(\mu_{t-(a+bS)})}{\partial \beta_0} + \sum_{a=1}^{q} \theta_a \frac{\partial^2 g(\mu_{t-a})}{\partial \beta_0 \partial \theta_a} +$$

$$\sum_{b=1}^{Q} \Theta_b \frac{\partial^2 g(\mu_{t-bS})}{\partial \beta_0 \partial \theta_a} - \sum_{a=1}^{q} \sum_{b=1}^{Q} \theta_a \Theta_b \frac{\partial^2 g(\mu_{t-(a+bS)})}{\partial \beta_0 \partial \theta_a},$$

$$\frac{\partial^2 g(\mu)}{\partial \beta_0 \partial \Phi_j} = \sum_{a=1}^{q} \theta_a \frac{\partial^2 g(\mu_{t-a})}{\partial \beta_0 \partial \Phi_j} + \sum_{b=1}^{Q} \Theta_b \frac{\partial^2 g(\mu_{t-bS})}{\partial \beta_0 \partial \Phi_j} - \sum_{a=1}^{q} \sum_{b=1}^{Q} \theta_a \Theta_b \frac{\partial^2 g(\mu_{t-(a+bS)})}{\partial \beta_0 \partial \Phi_j},$$

$$\frac{\partial^2 g(\mu_t)}{\partial \beta_0 \partial \Theta_b} = \frac{\partial}{\partial \beta_0} \left(\frac{\partial g(\mu_t)}{\partial \Theta_b} \right) = \frac{\partial g(\mu_{t-bS})}{\partial \beta_0} - \sum_{a=1}^{q} \theta_a \frac{\partial g(\mu_{t-(a+bS)})}{\partial \beta_0} + \sum_{a=1}^{q} \theta_a \frac{\partial^2 g(\mu_{t-a})}{\partial \beta_0 \partial \Theta_b} + \sum_{b=1}^{Q} \Theta_b \frac{\partial^2 g(\mu_{t-bS})}{\partial \beta_0 \partial \Theta_b} - \sum_{a=1}^{q} \sum_{b=1}^{Q} \theta_a \Theta_b \frac{\partial^2 g(\mu_{t-(a+bS)})}{\partial \beta_0 \partial \Theta_b},$$

$$\frac{\partial^2 g(\mu)}{\partial \beta_d \partial \beta_c} = \sum_{a=1}^{q} \theta_a \frac{\partial^2 g(\mu_{t-a})}{\partial \beta_d \partial \beta_c} + \sum_{b=1}^{Q} \Theta_b \frac{\partial^2 g(\mu_{t-bS})}{\partial \beta_d \partial \beta_c} - \sum_{a=1}^{q} \sum_{b=1}^{Q} \theta_a \Theta_b \frac{\partial^2 g(\mu_{t-(a+bS)})}{\partial \beta_d \partial \beta_c},$$

$$\frac{\partial^2 g(\mu_t)}{\partial \beta_c \partial \phi_i} = \frac{\partial}{\partial \beta_c} \left(\frac{\partial g(\mu)}{\partial \phi_i} \right) = -\mathbf{x}_{t-i,c} + \sum_{j=1}^{P} \Phi_j \mathbf{x}_{t-(i+jS),c} + \sum_{a=1}^{q} \theta_a \frac{\partial^2 g(\mu_{t-a})}{\partial \beta_c \partial \phi_i} + \sum_{b=1}^{Q} \Theta_b \frac{\partial^2 g(\mu_{t-bS})}{\partial \beta_c \partial \phi_i} - \sum_{a=1}^{q} \sum_{b=1}^{Q} \theta_a \Theta_b \frac{\partial^2 g(\mu_{t-(a+bS)})}{\partial \beta_c \partial \phi_i},$$

$$\frac{\partial^2 g(\mu_t)}{\partial \beta_c \partial \theta_a} = \frac{\partial}{\partial \beta_c} \left(\frac{\partial g(\mu_t)}{\partial \theta_a} \right) = \frac{\partial g(\mu_{t-a})}{\partial \beta_c} - \sum_{b=1}^{Q} \Theta_b \frac{\partial g(\mu_{t-(a+bS)})}{\partial \beta_c} + \sum_{a=1}^{q} \theta_a \frac{\partial^2 g(\mu_{t-a})}{\partial \beta_c \partial \theta_a} + \sum_{b=1}^{Q} \Theta_b \frac{\partial^2 g(\mu_{t-bS})}{\partial \beta_c \partial \theta_a} - \sum_{a=1}^{q} \sum_{b=1}^{Q} \theta_a \Theta_b \frac{\partial^2 g(\mu_{t-(a+bS)})}{\partial \beta_c \partial \theta_a},$$

$$\frac{\partial^2 g(\mu_t)}{\partial \beta_c \partial \Phi_j} = \frac{\partial}{\partial \beta_c} \left(\frac{\partial g(\mu)}{\partial \Phi_j} \right) = -\mathbf{x}_{t-jS,c} + \sum_{i=1}^{p} \phi_i \mathbf{x}_{t-(i+jS),c} + \sum_{a=1}^{q} \theta_a \frac{\partial^2 g(\mu_{t-a})}{\partial \beta_c \partial \Phi_j} + \sum_{b=1}^{Q} \Theta_b \frac{\partial^2 g(\mu_{t-bS})}{\partial \beta_c \partial \Phi_j} - \sum_{a=1}^{q} \sum_{b=1}^{Q} \theta_a \Theta_b \frac{\partial^2 g(\mu_{t-(a+bS)})}{\partial \beta_c \partial \Phi_j},$$

$$\frac{\partial^2 g(\mu_t)}{\partial \beta_c \partial \Theta_b} = \frac{\partial}{\partial \beta_c} \left(\frac{\partial g(\mu_t)}{\partial \Theta_b} \right) = \frac{\partial g(\mu_{t-bS})}{\partial \beta_c} - \sum_{a=1}^{q} \theta_a \frac{\partial g(\mu_{t-(a+bS)})}{\partial \beta_c} + \sum_{a=1}^{q} \theta_a \frac{\partial^2 g(\mu_{t-a})}{\partial \beta_c \partial \Theta_b} + \sum_{b=1}^{Q} \Theta_b \frac{\partial^2 g(\mu_{t-bS})}{\partial \beta_c \partial \Theta_b} - \sum_{a=1}^{q} \sum_{b=1}^{Q} \theta_a \Theta_b \frac{\partial^2 g(\mu_{t-(a+bS)})}{\partial \beta_c \partial \Theta_b},$$

$$\frac{\partial^2 g(\mu)}{\partial \phi_l \partial \phi_i} = \sum_{a=1}^{q} \theta_a \frac{\partial^2 g(\mu_{t-a})}{\partial \phi_l \partial \phi_i} + \sum_{b=1}^{Q} \Theta_b \frac{\partial^2 g(\mu_{t-bS})}{\partial \phi_l \partial \phi_i} - \sum_{a=1}^{q} \sum_{b=1}^{Q} \theta_a \Theta_b \frac{\partial^2 g(\mu_{t-(a+bS)})}{\partial \phi_l \partial \phi_i},$$

$$\frac{\partial^2 g(\mu_t)}{\partial \phi_i \partial \theta_a} = \frac{\partial}{\partial \phi_i} \left(\frac{\partial g(\mu_t)}{\partial \theta_a} \right) = \frac{\partial g(\mu_{t-a})}{\partial \phi_i} - \sum_{b=1}^{Q} \Theta_b \frac{\partial g(\mu_{t-(a+bS)})}{\partial \phi_i} + \sum_{a=1}^{q} \theta_a \frac{\partial^2 g(\mu_{t-a})}{\partial \phi_i \partial \theta_a} + \sum_{b=1}^{Q} \Theta_b \frac{\partial^2 g(\mu_{t-bS})}{\partial \phi_i \partial \theta_a} - \sum_{a=1}^{q} \sum_{b=1}^{Q} \theta_a \Theta_b \frac{\partial^2 g(\mu_{t-(a+bS)})}{\partial \phi_i \partial \theta_a},$$

$$\frac{\partial^2 g(\mu_t)}{\partial\phi_i\partial\Phi_j} = \frac{\partial}{\partial\phi_i}\left(\frac{\partial g(\mu_t)}{\partial\Phi_j}\right) = -\sum_{i=1}^{p}[g(y_{t-(i+jS)}) - \mathbf{x}_{t-(i+jS)}\boldsymbol{\beta}] + \sum_{a=1}^{q}\theta_a\frac{\partial^2 g(\mu_{t-a})}{\partial\phi_i\partial\Phi_j} + \sum_{b=1}^{Q}\Theta_b\frac{\partial^2 g(\mu_{t-bS})}{\partial\phi_i\partial\Phi_j} - \sum_{a=1}^{q}\sum_{b=1}^{Q}\theta_a\Theta_b\frac{\partial^2 g(\mu_{t-(a+bS)})}{\partial\phi_i\partial\Phi_j},$$

$$\frac{\partial^2 g(\mu_t)}{\partial\phi_i\partial\Theta_b} = \frac{\partial}{\partial\phi_i}\left(\frac{\partial g(\mu_t)}{\partial\Theta_b}\right) = \frac{\partial g(\mu_{t-bS})}{\partial\phi_i} - \sum_{a=1}^{q}\theta_a\frac{\partial g(\mu_{t-(a+bS)})}{\partial\phi_i} + \sum_{a=1}^{q}\theta_a\frac{\partial^2 g(\mu_{t-a})}{\partial\phi_i\partial\Theta_b} + \sum_{b=1}^{Q}\Theta_b\frac{\partial^2 g(\mu_{t-bS})}{\partial\phi_i\partial\Theta_b} - \sum_{a=1}^{q}\sum_{b=1}^{Q}\theta_a\Theta_b\frac{\partial^2 g(\mu_{t-(a+bS)})}{\partial\phi_i\partial\Theta_b},$$

$$\frac{\partial^2 g(\mu)}{\partial\theta_o\partial\theta_a} = \frac{\partial}{\partial\theta_o}\left(\frac{\partial g(\mu)}{\partial\theta_a}\right) = \frac{\partial g(\mu_{t-a})}{\partial\theta_o} - \sum_{b=1}^{Q}\Theta_b\frac{\partial g(\mu_{t-(a+bS)})}{\partial\theta_o} + \frac{\partial g(\mu_{t-o})}{\partial\theta_a} - \sum_{b=1}^{Q}\Theta_b\frac{\partial g(\mu_{t-(o+bS)})}{\partial\theta_a} + \sum_{a=1}^{q}\theta_a\frac{\partial^2 g(\mu_{t-a})}{\partial\theta_o\partial\theta_a} + \sum_{b=1}^{Q}\Theta_b\frac{\partial^2 g(\mu_{t-bS})}{\partial\theta_o\partial\theta_a} - \sum_{a=1}^{q}\sum_{b=1}^{Q}\theta_a\Theta_b\frac{\partial^2 g(\mu_{t-(a+bS)})}{\partial\theta_o\partial\theta_a},$$

$$\frac{\partial^2 g(\mu_t)}{\partial\theta_a\partial\Theta_b} = \frac{\partial}{\partial\theta_a}\left(\frac{\partial g(\mu_t)}{\partial\Theta_b}\right) = \frac{\partial g(\mu_{t-bS})}{\partial\theta_a} + r_{t-(a+bS)} - \sum_{a=1}^{q}\theta_a\frac{\partial g(\mu_{t-(a+bS)})}{\partial\theta_a} + \frac{\partial g(\mu_{t-a})}{\partial\Theta_b} - \sum_{b=1}^{Q}\Theta_b\frac{\partial g(\mu_{t-(a+bS)})}{\partial\Theta_b} + \sum_{a=1}^{q}\theta_a\frac{\partial^2 g(\mu_{t-a})}{\partial\theta_a\partial\Theta_b} + \sum_{b=1}^{Q}\Theta_b\frac{\partial^2 g(\mu_{t-bS})}{\partial\theta_a\partial\Theta_b} - \sum_{a=1}^{q}\sum_{b=1}^{Q}\theta_a\Theta_b\frac{\partial^2 g(\mu_{t-(a+bS)})}{\partial\theta_a\partial\Theta_b},$$

$$\frac{\partial^2 g(\mu_t)}{\partial\Phi_j\partial\theta_a} = \frac{\partial}{\partial\Phi_j}\left(\frac{\partial g(\mu_t)}{\partial\theta_a}\right) = \frac{\partial g(\mu_{t-a})}{\partial\Phi_j} - \sum_{b=1}^{Q}\Theta_b\frac{\partial g(\mu_{t-(a+bS)})}{\partial\Phi_j} + \sum_{a=1}^{q}\theta_a\frac{\partial^2 g(\mu_{t-a})}{\partial\Phi_j\partial\theta_a} + \sum_{b=1}^{Q}\Theta_b\frac{\partial^2 g(\mu_{t-bS})}{\partial\Phi_j\partial\theta_a} - \sum_{a=1}^{q}\sum_{b=1}^{Q}\theta_a\Theta_b\frac{\partial^2 g(\mu_{t-(a+bS)})}{\partial\Phi_j\partial\theta_a},$$

$$\frac{\partial^2 g(\mu)}{\partial\Phi_m\partial\Phi_j} = \sum_{a=1}^{q}\theta_a\frac{\partial^2 g(\mu_{t-a})}{\partial\Phi_m\partial\Phi_j} + \sum_{b=1}^{Q}\Theta_b\frac{\partial^2 g(\mu_{t-bS})}{\partial\Phi_m\partial\Phi_j} - \sum_{a=1}^{q}\sum_{b=1}^{Q}\theta_a\Theta_b\frac{\partial^2 g(\mu_{t-(a+bS)})}{\partial\Phi_m\partial\Phi_j},$$

$$\frac{\partial^2 g(\mu_t)}{\partial\Phi_j\partial\Theta_b} = \frac{\partial}{\partial\Phi_j}\left(\frac{\partial g(\mu_t)}{\partial\Theta_b}\right) = \frac{\partial g(\mu_{t-bS})}{\partial\Phi_j} - \sum_{a=1}^{q}\theta_a\frac{\partial g(\mu_{t-(a+bS)})}{\partial\Phi_j} + \sum_{a=1}^{q}\theta_a\frac{\partial^2 g(\mu_{t-a})}{\partial\Phi_j\partial\Theta_b} + \sum_{b=1}^{Q}\Theta_b\frac{\partial^2 g(\mu_{t-bS})}{\partial\Phi_j\partial\Theta_b} - \sum_{a=1}^{q}\sum_{b=1}^{Q}\theta_a\Theta_b\frac{\partial^2 g(\mu_{t-(a+bS)})}{\partial\Phi_j\partial\Theta_b},$$

$$\frac{\partial^2 g(\mu)}{\partial\Theta_v\partial\Theta_b} = \frac{\partial}{\partial\Theta_v}\left(\frac{\partial g(\mu)}{\partial\Theta_b}\right) = \frac{\partial g(\mu_{t-bS})}{\partial\Theta_v} - \sum_{a=1}^{q}\theta_a\frac{\partial g(\mu_{t-(a+bS)})}{\partial\Theta_v} + \frac{\partial g(\mu_{t-vS})}{\partial\Theta_b} - \sum_{a=1}^{q}\theta_a\frac{\partial g(\mu_{t-(a+vS)})}{\partial\Theta_b} + \sum_{a=1}^{q}\theta_a\frac{\partial^2 g(\mu_{t-a})}{\partial\Theta_v\partial\Theta_b} +$$

$$\sum_{b=1}^{Q}\Theta_b\frac{\partial^2 g(\mu_{t-bS})}{\partial\Theta_v\partial\Theta_b}-\sum_{a=1}^{q}\sum_{b=1}^{Q}\theta_a\Theta_b\frac{\partial^2 g(\mu_{t-(a+bS)})}{\partial\Theta_v\partial\Theta_b}.$$
