# Supplementary material for: Modified Kumaraswamy seasonal autoregressive moving average models with exogenous regressors for double-bounded hydro-environmental data
Source: PLoS One. 2025 May 20;20(5):e0324721. doi: 10.1371/journal.pone.0324721 (PMC12091792; doi:10.1371/journal.pone.0324721)
Supplement: S2 Appendix — (PDF) [file pone.0324721.s002.pdf]

S2 Appendix.

Supplementary simulation results

In this appendix, we provide additional simulation results to complement our analysis. Specifically, we present the simulation results of MKSARMA $(1,0) \times (1,0)_{12}$ scenario in Table 1, and of MKSARMA $(0,1) \times (0,1)_{12}$ scenario in Table 2. These results further elucidate our proposed model parameter estimation performance under different scenarios and parameter configurations.

Table 1. Mean, bias, relative bias, standard error, and MSE of MKSARMA $(\mathbf{1},\mathbf{0}) \times (\mathbf{1},\mathbf{0})_{\mathbf{12}}$ parameter estimators, and coverage rate for the confidence interval. Parameter values are presented inside the parentheses.

**Measures**	$\boldsymbol{\beta_0}$ **(0.5290)**	$\boldsymbol{\phi_1}$ **(0.4229)**	$\boldsymbol{\Phi_1}$ **(0.2971)**	$\boldsymbol{\alpha}$ **(15.6063)**
Sample size = 100				
Mean	0.5499	0.4094	0.2871	16.1311
Bias	0.0209	−0.0135	−0.0100	0.5248
Bias (%)	3.9496	3.1870	3.3509	3.3630
Standard error	0.1042	0.0874	0.0927	1.4731
MSE	0.0113	0.0078	0.0087	2.4455
Coverage rate	0.9470	0.9492	0.9480	0.9384
Sample size = 300				
Mean	0.5351	0.4194	0.2928	15.7280
Bias	0.0061	−0.0035	−0.0043	0.1217
Bias (%)	1.1458	0.8200	1.4350	0.7800
Standard error	0.0524	0.0456	0.0482	0.7620
MSE	0.0028	0.0021	0.0023	0.5955
Coverage rate	0.9484	0.9498	0.9452	0.9482
Sample size = 500				
Mean	0.5325	0.4204	0.2953	15.6971
Bias	0.0035	−0.0025	−0.0018	0.0908
Bias (%)	0.6682	0.5820	0.5987	0.5821
Standard error	0.0397	0.0348	0.0367	0.5962
MSE	0.0016	0.0012	0.0013	0.3637
Coverage rate	0.9484	0.9470	0.9482	0.9460
Sample size = 700				
Mean	0.5316	0.4214	0.2957	15.6776
Bias	0.0026	−0.0015	−0.0014	0.0713
Bias (%)	0.4992	0.3615	0.4562	0.4566
Standard error	0.0333	0.0292	0.0301	0.4874
MSE	0.0011	0.0009	0.0009	0.2427
Coverage rate	0.9492	0.9476	0.9488	0.9478

Table 2. Mean, bias, relative bias, standard error, and MSE of MKSARMA $(\mathbf{0},\mathbf{1})\times(\mathbf{0},\mathbf{1})_{\mathbf{12}}$ parameter estimators, and coverage rate for the confidence interval. Parameter values are presented inside the parentheses.

**Measures**	$\boldsymbol{\beta_0}$ **(1.2924)**	$\boldsymbol{\theta_1}$ $(-\mathbf{0.3380})$	$\boldsymbol{\Theta_1}$ $(-\mathbf{0.3747})$	$\boldsymbol{\alpha}$ **(14.9843)**
Sample size = 100				
Mean	1.2957	−0.3375	−0.3659	15.2152
Bias	0.0033	0.0005	0.0088	0.2309
Bias (%)	0.2561	0.1342	2.3383	1.5406
Standard error	0.0552	0.0957	0.0994	1.3971
MSE	0.0031	0.0092	0.0100	2.0053
Coverage rate	0.9286	0.9216	0.9326	0.9424
Sample size = 300				
Mean	1.2928	−0.3384	−0.3716	15.0132
Bias	0.0004	−0.0004	0.0031	0.0289
Bias (%)	0.0317	0.1285	0.8313	0.1927
Standard error	0.0306	0.0468	0.0473	0.7278
MSE	0.0009	0.0022	0.0023	0.5305
Coverage rate	0.9456	0.9436	0.9434	0.9498
Sample size = 500				
Mean	1.2928	−0.3381	−0.3737	15.0178
Bias	0.0004	−0.0001	0.0010	0.0335
Bias (%)	0.0302	0.0354	0.2677	0.2234
Standard error	0.0234	0.0358	0.0355	0.5731
MSE	0.0005	0.0013	0.0013	0.3295
Coverage rate	0.9474	0.9454	0.9450	0.9450
Sample size = 700				
Mean	1.2932	−0.3376	−0.3749	15.0191
Bias	0.0008	0.0004	−0.0002	0.0348
Bias (%)	0.0622	0.1263	0.0433	0.2325
Standard error	0.0197	0.0296	0.0295	0.4695
MSE	0.0004	0.0009	0.0009	0.2216
Coverage rate	0.9498	0.9478	0.9480	0.9500
